# Supramolecular Assemblies of Dipyrrolyldiketone Cu^II^ Complexes

**DOI:** 10.3390/molecules26040861

**Published:** 2021-02-06

**Authors:** Yohei Haketa, Hiromitsu Maeda

**Affiliations:** Department of Applied Chemistry, College of Life Sciences, Ritsumeikan University, Kusatsu 525-8577, Japan; yhaketa@fc.ritsumei.ac.jp

**Keywords:** π-electronic systems, dipyrrolyldiketones, Cu^II^ complexes, crystal structures, mesophases

## Abstract

Dipyrrolyldiketones are essential building units of anion-responsive π-electronic molecules and ion-pairing assemblies. Here, we demonstrated that they form complexes with Cu^II^ characterized by planar geometries. The solid-state stacking assembled structures, as revealed by single-crystal X-ray analysis, were modulated by the substitution of pyrrole units. The rectangular shapes of the Cu^II^ complexes resulted in the formation of mesophases upon introduction of aliphatic chains.

## 1. Introduction

**Figure 1:**
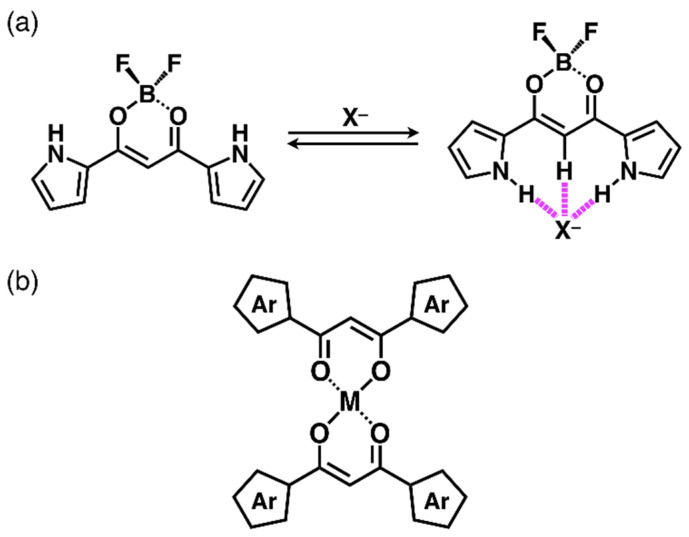
(**a**) Dipyrrolyldiketone BF_2_ complex and its anion-binding mode and (**b**) 1,3-diketone divalent metal complexes.

The fabrication of molecular assemblies is crucial for the development of functional materials [1]. In particular, π-electronic molecules with planar geometries may afford stacking assemblies, which are suitable for the generation of electrically conductive materials. Ion-pairing assemblies based on π-electronic ions form a variety of ordered states that act as dimension-controlled assemblies, such as crystals, liquid crystals, and supramolecular gels [2,3]. The preparation of π-electronic anions, which can serve as components of ion-pairing assemblies, is more difficult than that of π-electronic cations due to the lower stability of electron-rich species. Therefore, anion-responsive π-electronic molecules have been investigated for the preparation of anion complexes which are pseudo π-electronic anions. For example, boron complexes of dipyrrolyldiketones (Figure 1a) provided planar anion complexes through hydrogen-bonding interactions with pyrrole-NH and bridging-CH units, which were used as components of various ion-pairing assemblies [4,5,6,7,8]. Anion binding requires the inversion of two pyrrole rings from the state where the NH units are oriented towards the carbonyl units, as the most stable conformations generally result from the opposite dipole arrangements of the pyrrole rings and carbonyl units.

Notably, the 1,3-diketone unit can act as a monovalent metal ligand for various metal complexes (Figure 1b) [9,10,11,12,13,14,15,16]. The complexation of 1,3-diketones with divalent metals, such as Cu^II^, Ni^II^, and Pt^II^, formed planar geometries, which are suitable for the formation of stacking assemblies. For example, various liquid-crystal materials based on 1,3-diketone metal complexes were fabricated using aryl units that served as scaffolds for aliphatic chains [11,12,13,14,15,16]. On the other hand, the introduction of divalent metals to dipyrrolyldiketones were not achieved due to the coordination by the pyrrole-N sites. Therefore, appropriate metal complexation conditions are required for the development of dipyrrolyldiketone metal complexes. Recently, Ti^IV^ complexes of dipyrrolyldiketones were investigated by focusing on their crystal and liquid-crystal mesophase structures [17], prompting further investigations on other metal complexes [18]. This study describes the synthesis of Cu^II^ complexes of dipyrrolyldiketones, as well as the investigation of their crystal structures and mesophase assemblies.

## 2. Results and Discussion

**Figure 2:**
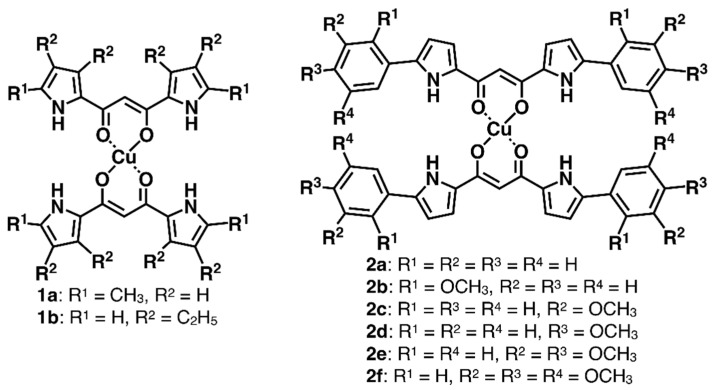
Dipyrrolyldiketone Cu^II^ complexes **1a**,**b** and **2a**–**f**. The pyrrole positions bearing R^1^ and R^2^ in **1a**,**b** are α and β, respectively, and aryl rings are substituted at the α-positions in **2a**–**f**.

Dipyrrolyldiketone Cu^II^ complexes **1a**,**b** and **2a**–**f** (Figure 2) were synthesized according to the literature procedures, by treating dipyrrolyldiketones **1a′**,**b′** and **2a′**–**f′** [19,20,21,22] with Cu(OAc)_2_ in MeOH/CHCl_3_ [11]; moderate yields were obtained depending on the purification process. Due to the low stability of the 1,3-diketone–Cu coordination bonds under silica gel purification conditions, **1a**,**b** and **2a**–**f** were isolated by reprecipitation. The ^1^H NMR of **1a**,**b** and **2a**–**f** displayed broad signals due to the paramagnetic properties of Cu^II^ as d^9^ electron configuration. Therefore, **1a**,**b** and **2a**–**f** were characterized by MALDI-TOF- and ESI-TOF-MS analysis. The UV/vis absorption maximum (λ_max_) of **1a** as a representative example in CH_2_Cl_2_ was 399 nm, which can be attributed to the transition at the π-electronic systems, as suggested by time-dependent (TD)-DFT calculations at the B3LYP/6-31G(d,p) level with LanL2DZ for Cu [23].

**Figure 3:**
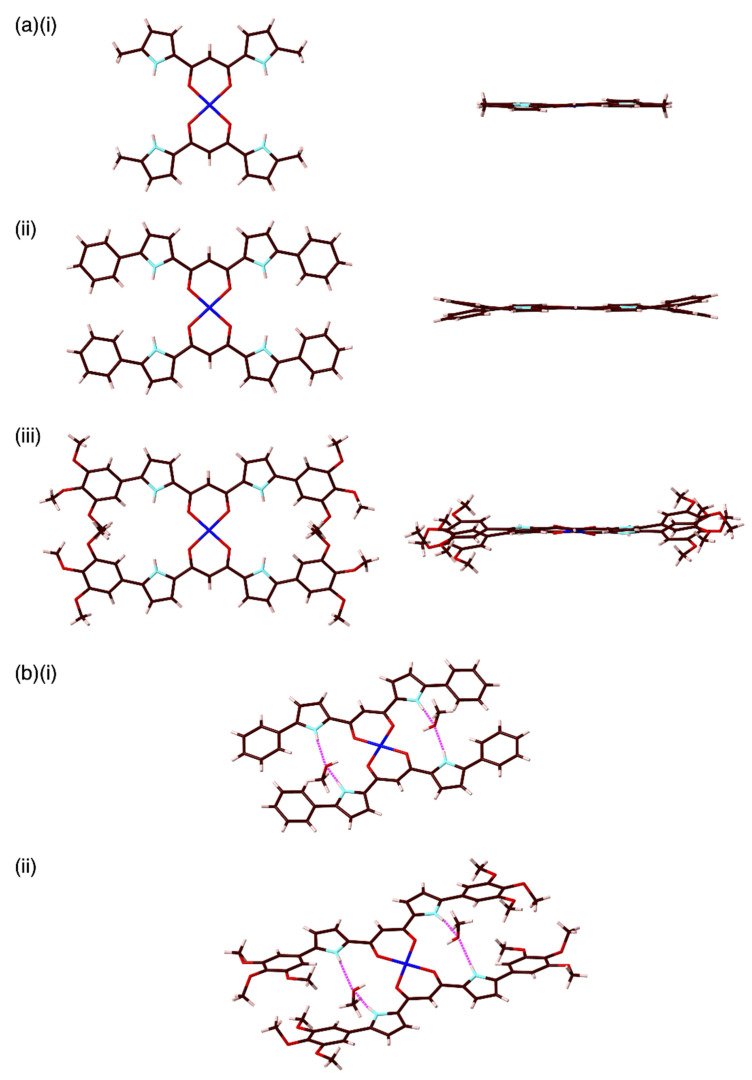
Molecular structures of dipyrrolyldiketone Cu^II^ complexes revealed by the single-crystal X-ray analysis as (**a**) top and side views ((i) **1a**, (ii) **2a**, and (iii) **2f** and (**b**) MeOH-binding structures for (i) **2a** (one of the two independent structures) and (ii) **2f**. Color code: Brown, pink, cyan, red, and blue refer to carbon, hydrogen, nitrogen, oxygen, and copper, respectively.

Single crystals of **1a**,**b** and **2a**–**c**,**f** for X-ray analysis were obtained using the vapor diffusion technique according to the solvent-dependent solubilities (see the Supplementary Materials for details). The Cu atoms in all the structures except for **2c** lie on special positions. In the solid state, **1a** and **2a**–**c**,**f** exhibited planar geometries for the dipyrrolyldiketone Cu^II^ unit with mean-plane deviations of 0.065(2) Å for **1a** (core dipyrrolyldiketone Cu^II^ unit as 31 atoms) and 0.041(4)/0.056(7), 0.127(4), 0.029(2), and 0.091(2) Å for **2a**–**c**,**f** (core dipyrrolyldiketone Cu^II^ and aryl units as 55 atoms), respectively (Figure 3a; see Figures S14, S16 and S17  for **1b** and **2b**,**c**). On the other hand, **1b** showed a distorted structure with a dihedral angle of 29.86(5)° for two oppositely arranged diketone mean planes (15 atoms of the two pyrrole and core diketone units). In each case, two pyrrole-NH units were oriented to the Cu^II^ (carbonyl) side as the most stable conformation, which was also revealed by DFT calculations [23]. For example, **1a** with uninverted pyrrole rings was more stabilized by 17.62 kcal/mol than the structure with completely inverted pyrrole rings, as suggested by DFT calculations at the B3LYP/6-31G(d,p) level with LanL2DZ for Cu. The antiparallel direction of the dipoles for the pyrrole and carbonyl moieties was a key factor for determining the planar structure. The C_carbonyl_–C_bridging_–C_carbonyl_ angles for **1a**,**b** and **2a**–**c**,**f** were found to be 123.5(8)–126.4(2)°, which are similar to those of other 1,3-diketone Cu^II^ complexes [11,12]. Furthermore, the O–Cu–O angles were in the 93.3(5)–94.9(3)° range, which are typical values for divalent metal 1,3-diketone complexes. Similar O–Cu–O angles were observed by DFT calculations at the B3LYP/6-31G(d,p) level with LanL2DZ for Cu. The τ_4_ parameters [24], which indicate the geometries of four-coordinate transition metal complexes, also showed square planar structures of the 1,3-diketone Cu^II^ complex units in **1a**,**b** and **2a**–**c**,**f**. The substituted aryl units in **2a**–**c** were slightly twisted in the crystal state with dihedral angles of 4.4(2)–10.7(5)°, which were smaller than those of the phenyl-substituted dipyrrolyldiketone BF_2_ complex (26.0 and 19.4°) [21]. On the other hand, 3,4,5-trimethoxyphenyl-substituted **2f** displayed larger dihedral angles (18.5(1) and 23.4(1)°) for the pyrrole and aryl units due to the steric repulsion between the *m*-methoxy groups of the dipyrrolyldiketone units. Interestingly, in the case of **1a**, two water molecules were bound in the cavities through hydrogen-bonding interactions with the pyrrole NH, exhibiting N(–H)···O distances of 2.88(1) and 2.90(1) Å. Similarly, in the case of **2a**–**c**,**f**, the crystallization solvent MeOH was bound in the cavities surrounded by two pyrrole-NH and aryl-*o*-CH (Figure 3b). For example, in the case of **2f**, the pyrrole-N(–H)···O and aryl-*o*-C(–H)···O distances were 2.86(1)/2.91(1) and 3.495(4)/3.87(1) Å, respectively, indicating the occurrence of hydrogen-bonding interactions. Smaller dihedral angles between the pyrrole and aryl rings resulted from the hydrogen bonding of the pyrrole-NH and aryl-*o*-CH with MeOH. The guest-binding cavities with pyrrole-NH and aryl-*o*-CH sites are characteristic of the Cu^II^ complexes of aryl-substituted dipyrrolyldiketones.

**Figure 4:**
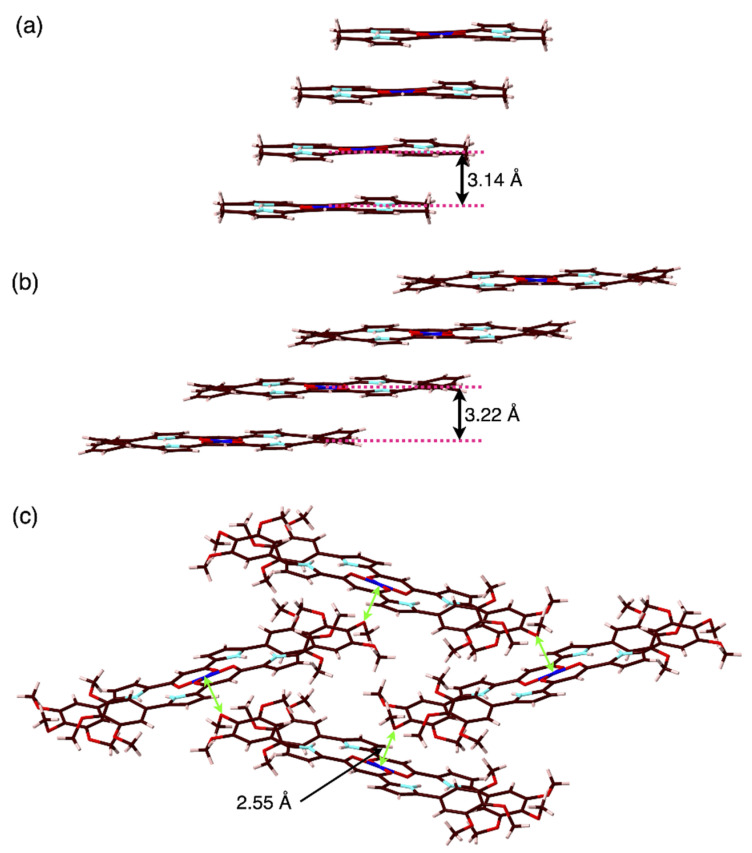
Single-crystal X-ray structures of dipyrrolyldiketone Cu^II^ complexes (**a**) **1a**, (**b**) **2a** (one of the two independent structures), and (**c**) **2f** shown as packing diagrams with, in (**c**), green arrows indicating the coordination between the *p*-methoxy oxygen and Cu^II^. Solvent molecules are omitted for clarity. Color code: Brown, pink, cyan, red, and blue refer to carbon, hydrogen, nitrogen, oxygen, and copper, respectively.

**Figure 5:**
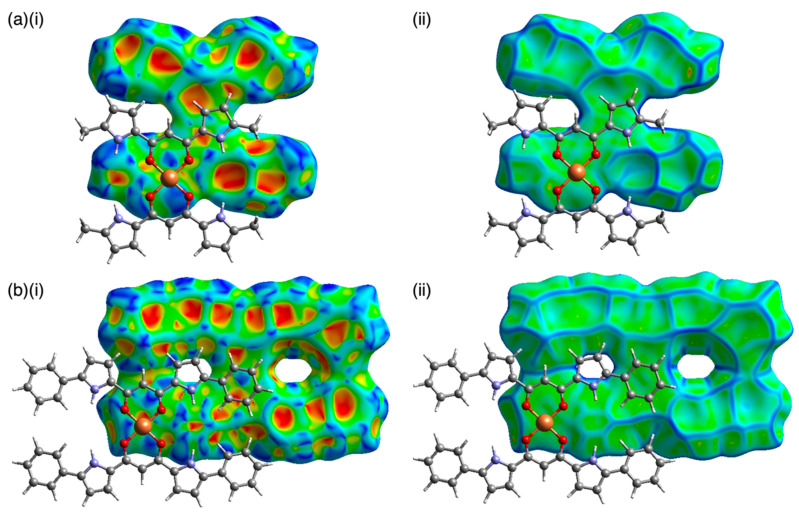
Hirshfeld surfaces of dipyrrolyldiketone Cu^II^ complexes (**a**) **1a** and (**b**) **2a** as two stacking molecules ((i) mapped over shape-index and (ii) curvedness properties). Solvent molecules are omitted for clarity. Color code: Gray, white, blue, red, and orange refer to carbon, hydrogen, nitrogen, oxygen, and copper, respectively.

Owing to the core planar geometries of dipyrrolyldiketone Cu^II^ complexes, slipped-stacking structures were observed in the crystal states. For example, **1a** showed a slipped-stacking columnar structure with a stacking distance of 3.14 Å between the two mean planes of the core dipyrrolyldiketone Cu^II^ unit (31 atoms of two dipyrrolyldiketone moieties and Cu) (Figure 4a). On the other hand, the distorted **1b** showed a packing structure with no stacking, probably due to the steric repulsion between the β-ethyl units. Interestingly, **1b** formed a hydrogen-bonding 1D-columnar structure based on the interaction of the pyrrole NH and the neighboring oxygen of the diketone unit (Figure S14). Similar to **1a**, aryl-substituted **2a**–**c** formed slipped-stacking columnar structures with stacking distances of 3.24–3.22 Å between the two mean planes of the aryl-substituted dipyrrolyldiketone Cu^II^ complex unit (55 atoms) (Figure 4b). The Cu^II^···Cu^II^ distances in the stacking structures of **1a** and **2a** were 5.901(3) and 9.08(2) Å, respectively. Furthermore, angles of 32.1 and 19.8° were observed along the lines passing through two Cu^II^ in the columnar structures of **1a** and **2a** to the mean planes of the core dipyrrolyldiketone Cu^II^ complex units (31 atoms), respectively. The introduction of aryl units at the α-positions extended the planar structures to the long axis of the molecules, and thus, the aryl rings stacked on top of the diketone Cu^II^ planes. In order to visualize the intermolecular interactions in the crystal structures, the Hirshfeld surface analysis was carried out [25,26,27]. In fact, the Hirshfeld surface analysis of **1a** and **2a** revealed an effective stacking of the core 1,3-diketone Cu^II^ complex unit with the pyrrole and phenyl rings, respectively (Figure 5). In particular, the surfaces of the phenyl rings of **2a** showed the red and blue triangles arranged in bow-tie shapes on the shape-index surface and flat region on the curvedness surface, indicating the characteristic mapping pattern for the stacking of planar units including aryl rings [28]. The stacking structures of the dipyrrolyldiketone Cu^II^ complexes as shown here were also stabilized by the chelate ring–π interaction [29]. The rectangular geometries formed by the introduction of aryl units are crucial for modulating the assembled structures. In contrast, 3,4,5-trimethoxy-substituted **2f** exhibited a zigzag-arranged packing structure. The *p*-methoxy oxygen was coordinated to Cu^II^ of the neighboring complex with an O···Cu^II^ distance of 2.547(8) Å (Figure 4c).

**Figure 6:**
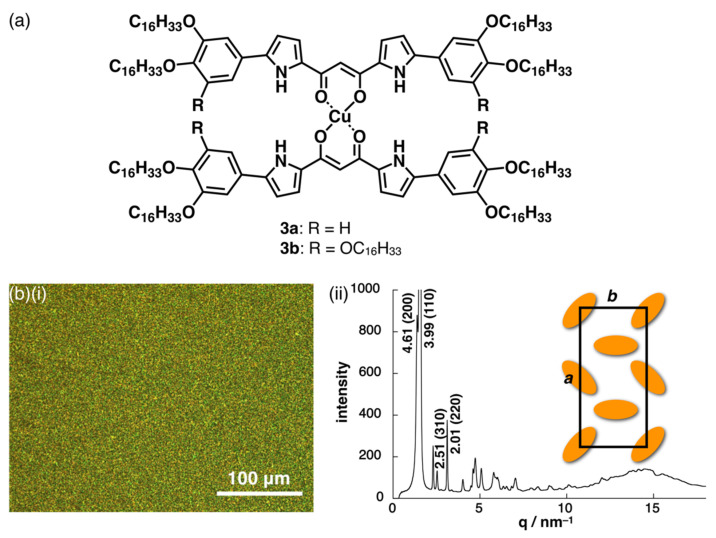
(**a**) Dipyrrolyldiketone Cu^II^ complexes **3a**,**b** and (**b**)(i) POM observation and (ii) XRD analysis, with a packing model, of **3a** at 90 °C upon cooling.

On the basis of the rectangular discotic geometries of the dipyrrolyldiketone Cu^II^ complexes, the formation of dimension-controlled assemblies as mesophases was investigated. To induce mesophases, 3,4-dihexadecyloxy and 3,4,5-trihexadecyloxy chains were introduced at the aryl rings of **3a**,**b** (Figure 6a). Differential scanning calorimetry (DSC) analysis of dihexadecyloxy **3a** revealed the formation of mesophases with transition temperatures of 104/86/36 and 28/96/107 °C upon cooling and heating, respectively, and a mosaic polarized optical microscopy (POM) texture (Figure 6b(i)). On the other hand, **3b** exhibited broad transitions at the temperatures at 49/37/24 and 27/43/50 °C upon cooling and heating, respectively. X-ray diffraction (XRD) analysis of **3a** at 90 °C upon cooling revealed peaks at 4.61, 3.99, 2.51, and 2.01 nm, which were indexed to (200), (110), (310), and (220), respectively, suggesting the formation of a Col_r_ (*P*2/*a*) structure with *a* = 9.22 nm, *b* = 4.43 nm, *c* = 0.39 nm, and *Z* = 4 (*ρ* = 1.1) (Figure 6b(ii)) [30]. It should be noted that dipyrrolyldiketone Cu^II^ complexes possessed rectangular geometries as the most stable structures based on the pyrrole NH directed toward the carbonyl moieties. In contrast to the columnar structure of **3a**, trihexadecyloxy **3b** exhibited XRD peaks at 6.77, 3.35, and 2.30 nm, which were indexed to (001), (002), and (003), respectively, suggesting the formation of a discotic lamellar structure with an interlayer distance of 6.77 nm. The length of **3b** was estimated to be ca. 6 nm, which is in good agreement with the speculated packing structure formed by interdigitating the aliphatic chains.

## 3. Materials and Methods

### 3.1. Synthetic Procedures and Spectroscopic Data

#### 3.1.1. General Procedures

Starting materials were purchased from FUJIFILM Wako Pure Chemical Corp. (Osaka, Japan), Nacalai Tesque Inc. (Kyoto, Japan), Tokyo Chemical Industry Co., Ltd. (Tokyo, Japan), and Sigma-Aldrich Co. (Tokyo, Japan) and were used without further purification unless otherwise stated. NMR spectra used in the characterization of precursors of Cu^II^ complexes were recorded on a JEOL ECA-600 600 MHz spectrometer (JEOL Ltd., Tokyo, Japan). UV-visible absorption spectra were recorded on a Hitachi U-3500 spectrometer (Hitachi High-Tech Science Corp., Tokyo, Japan). Matrix-assisted laser desorption ionization time-of-flight mass spectrometries (MALDI-TOF-MS) were recorded on a Shimadzu Axima-CFRplus (Shimadzu Corp., Kyoto, Japan). High-resolution (HR) electrospray ionization mass spectrometries (ESI-MS) were recorded on a BRUKER microTOF using the ESI-TOF method (Bruker, MA, USA). TLC analyses were carried out on aluminum sheets coated with silica gel 60 (Merck 5554). Column chromatography was performed on Wakogel C-300 and Merck silica gel 60H.

#### 3.1.2. 1,3-Bis(5-(3,4-dimethoxyphenyl)pyrrol-2-yl)-1,3-propanedione (2e′)

A CH_2_Cl_2_ (20 mL) solution of 2-(3,4-dimethoxyphenyl)pyrrole (276.9 mg, 1.36 mmol) [31] was treated with malonyl chloride (115.01 mg, 0.816 mmol) at room temperature and was stirred for 1 h at the same temperature. After confirming the consumption of the starting pyrrole by the TLC analysis, the mixture was washed with saturated aqueous Na_2_CO_3_ and water, dried over anhydrous MgSO_4_, filtered, and evaporated to dryness. The residue was then chromatographed over a silica gel column (eluent: 2% MeOH/CH_2_Cl_2_) and was recrystallized from CH_2_Cl_2_/*n*-hexane to afford **2e′** (166.2 mg, 0.35 mmol, 51%) as a pale-yellow solid. *R*_f_ = 0.27 (2% MeOH/CH_2_Cl_2_). ^1^H NMR (600 MHz, CDCl_3_, 20 °C; the diketone is obtained as a mixture of keto and enol tautomers in the ratio of 1:0.34): δ (ppm) keto form 9.43 (br, 2H, NH), 7.15–7.14 (m, 4H, pyrrole-H and Ar-H), 7.04 (m, 2H, Ar-H), 6.92–6.90 (m, 2H, Ar-H), 6.51–6.50 (m, 2H, pyrrole-H), 4.24 (s, 2H, CH_2_), 3.96–3.91 (m, 12H, OCH_3_); enol form 16.76 (br, 1H, OH), 9.35 (s, 2H, NH), 7.15–7.14 (m, 2H, Ar-H), 7.06 (m, 2H, Ar-H), 6.97–6.96 (m, 2H, pyrrole-H), 6.93–6.92 (m, 2H, Ar-H), 6.55–6.54 (m, 2H, pyrrole-H), 6.36 (s, 1H, CH), 3.96–3.91 (m, 12H, OCH_3_). MALDI-TOF-MS *m*/*z* (% intensity): 473.2 (100). Calcd for C_27_H_25_N_2_O_6_ ([M − H]^−^): 473.17.

#### 3.1.3. 1-tert-Butoxycarbonyl-2-(3,4-dihexadecyloxyphenyl)pyrrole and 2-(3,4-dihexadecyloxyphenyl)pyrrole

To a solution of 5-bromo-1,2-dihexadecyloxybenzene [32] (2.93 g, 4.60 mmol), 1-*tert*-butoxycarbonylpyrrole-2-boronic acid (1.0 g, 4.74 mmol), and Pd(PPh_3_)_4_ (543 mg, 0.47 mmol) in 1,2-dimethoxyethane (30 mL) at room temperature under nitrogen, a solution of Na_2_CO_3_ (2.5 g, 24.0 mmol) was added in water (3 mL). The mixture was heated at reflux temperature for 4 h, cooled, and then partitioned between water and CH_2_Cl_2_. The combined extracts were dried over anhydrous MgSO_4_ and evaporated to give a solid. The residue was then chromatographed over a flash silica gel column (eluent: 3% EtOAc/*n*-hexane) to give 1-*tert*-butoxycarbonyl-2-(3,4-dihexadecyloxyphenyl)pyrrole (2.83 g, 3.90 mmol, 85%) as a white solid. *R*_f_ = 0.32 (eluent: 3% EtOAc/*n*-hexane). ^1^H NMR (600 MHz, CDCl_3_, 20 °C): δ (ppm) 7.32–7.31 (m, 1H, pyrrole-H), 6.87–6.84 (s, 2H, Ar-H), 6.85–6.84 (m, 1H, Ar-H), 6.21–6.20 (m, 1H, pyrrole-H), 6.15–6.14 (m, 1H, pyrrole-H), 4.00 (t, *J* = 6.6 Hz, 2H, OCH_2_), 3.98 (t, *J* = 6.6 Hz, 2H, OCH_2_), 1.84–1.78 (m, 4H, OCH_2_C*H*_2_), 1.49–1.42 (m, 4H, OC_2_H_4_C*H*_2_), 1.37 (s, 9H, Boc), 1.35–1.25 (m, 48H, OC_3_H_6_C_12_*H*_24_), 0.89–0.86 (m, 6H, OC_15_H_30_C*H*_3_). MALDI-TOF-MS: *m*/*z* (% intensity) 723.7 (100). Calcd for C_47_H_81_NO_4_ ([M]^+^): 723.62. The produced 1-*tert*-butoxycarbonyl-2-(3,4-dihexadecyloxyphenyl)pyrrole (2.0 g, 2.76 mmol) was heated at 180 °C for 30 min. The residue was then chromatographed over a flash silica gel column (eluent: CH_2_Cl_2_/*n*-hexane = 1/1) to give 2-(3,4-dihexadecyloxyphenyl)pyrrole as a white solid (1.50 g, 2.40 mmol, 87%). *R*_f_ = 0.29 (CH_2_Cl_2_/*n*-hexane = 1/1). ^1^H NMR (600 MHz, CDCl_3_, 20 °C): δ (ppm) 8.32 (br, 1H, NH), 6.91 (d, *J* = 1.8 Hz, 1H, Ar-H), 6.98 (dd, *J* = 8.4 and 2.4 Hz, 1H, Ar-H), 6.88 (d, *J* = 8.4 Hz, 1H, Ar-H), 6.83–6.82 (m, 1H, pyrrole-H), 6.40–6.39 (m, 1H, pyrrole-H), 6.28–6.27 (m, 1H, pyrrole-H), 4.03 (t, *J* = 6.0 Hz, 2H, OCH_2_), 4.00 (t, *J* = 6.6 Hz, 2H, OCH_2_), 1.85–1.79 (m, 4H, OCH_2_C*H*_2_), 1.50–1.44 (m, 4H, OC_2_H_4_C*H*_2_), 1.37–1.33 (m, 4H, OC_3_H_6_C*H*_2_), 1.31–1.25 (m, 44H, OC_4_H_8_C_11_*H*_22_), 0.89–0.87 (m, 6H, OC_15_H_30_C*H*_3_). MALDI-TOF-MS: *m*/*z* (% intensity): 624.5 (100). Calcd for C_42_H_74_NO_2_ ([M + H]^+^): 624.57.

#### 3.1.4. 1,3-Bis(5-(3,4-dihexadecyloxyphenyl)pyrrol-2-yl)-1,3-propanedione (3a′)

A CH_2_Cl_2_ (40 mL) solution of 2-(3,4-dihexadecyloxyphenyl)pyrrole (1.0 g, 1.6 mmol) was treated with malonyl chloride (124.2 mg, 0.881 mmol) at room temperature and stirred at reflux temperature for 1 h. After confirming the consumption of the starting pyrrole by TLC, the mixture was washed with saturated aqueous Na_2_CO_3_ and water, dried over anhydrous MgSO_4_, filtered, and evaporated to dryness. The residue was then chromatographed over a silica gel column (eluent: CHCl_3_) and was reprecipitated from CHCl_3_/MeOH to afford **3a′** (441.8 mg, 0.34 mmol, 42%) as a pale-yellow solid. *R*_f_ = 0.25 (CHCl_3_). ^1^H NMR (600 MHz, CDCl_3_, 20 °C; the diketone is obtained as a mixture of keto and enol tautomers in the ratio of 1:0.24): δ (ppm) keto form 9.36 (br, 2H, NH), 7.14–7.13 (m, 2H, pyrrole-H), 7.12–7.09 (m, 2H, Ar-H), 7.04 (m, 2H, Ar-H), 6.90–6.88 (m, 2H, Ar-H), 6.49–6.48 (m, 2H, pyrrole-H), 4.22 (s, 2H, CH_2_), 4.06–4.00 (m, 8H, OCH_2_), 1.86–1.80 (m, 8H, OCH_2_C*H*_2_), 1.51–1.44 (m, 8H, OC_2_H_4_C*H*_2_), 1.36–1.34 (m, 8H, OC_3_H_6_C*H*_2_), 1.30–1.25 (m, 88H, OC_4_H_8_C_11_*H*_22_), 0.89–0.86 (m, 18H, OC_15_H_30_C*H*_3_); enol form 16.77 (br, 1H, OH), 9.29 (s, 2H, NH), 7.12 (m, 2H, Ar-H), 7.07 (m, 2H, Ar-H), 6.96–6.94 (m, 2H, pyrrole-H), 6.92–6.90 (m, 2H, Ar-H), 6.52–6.51 (m, 2H, pyrrole-H), 6.34 (s, 1H, CH), 4.06–4.00 (m, 8H, OCH_2_), 1.86–1.80 (m, 8H, OCH_2_C*H*_2_), 1.51–1.44 (m, 8H, OC_2_H_4_C*H*_2_), 1.36–1.34 (m, 8H, OC_3_H_6_C*H*_2_), 1.30–1.25 (m, 88H, OC_4_H_8_C_11_*H*_22_), 0.89–0.86 (m, 18H, OC_15_H_30_C*H*_3_). MALDI-TOF-MS *m*/*z* (% intensity): 1314.1 (100). Calcd for C_87_H_145_N_2_O_6_ ([M − H]^−^): 1314.11.

#### 3.1.5. Cu^II^ complex of 1,3-di(5-methylpyrrol-2-yl)-1,3-propanedione (1a)

A mixture of 1,3-di(5-methylpyrrol-2-yl)-1,3-propanedione **1a′** [33] (11.8 mg, 0.051 mmol) and Cu(OAc)_2_ (4.6 mg, 0.025 mmol) in MeOH (50 mL) was stirred at 25 °C for 1 h. After the solvent was evaporated, the residue was recrystallized from MeOH/*n*-hexane to afford **1a** (4.52 mg, 8.7 μmol, 34%) as a brown solid. UV/vis (CH_2_Cl_2_, λ_max_[nm] (ε, 10^4^ M^−1^cm^−1^)): 399 (7.5). MALDI-TOF-MS: *m*/*z* (% intensity): 520.1 (100). Calcd for C_26_H_25_CuN_4_O_4_ ([M − H]^−^): 520.12. HRMS (ESI-TOF): *m*/*z* 520.1177. Calcd for C_26_H_25_CuN_4_O_4_ ([M − H]^−^]): 520.1177. This compound was further characterized by the X-ray diffraction analysis.

#### 3.1.6. Cu^II^ Complex of 1,3-bis(3,4-diethylpyrrol-2-yl)-1,3-propanedione (1b)

A mixture of 1,3-bis(3,4-diethylpyrrol-2-yl)-1,3-propanedione **1b′ [20]** (15.8 mg, 0.050 mmol) and Cu(OAc)_2_ (4.6 mg, 0.025 mmol) in MeOH (50 mL) was stirred at 25 °C for 1 h. After the solvent was evaporated, the residue was recrystallized from MeOH/*n*-hexane to afford **1b** (7.24 mg, 10.5 μmol, 42%) as a brown solid. UV/vis (CH_2_Cl_2_, λ_max_[nm] (ε, 10^4^ M^−1^cm^−1^)): 393 (6.0). MALDI-TOF-MS: *m/z* (% intensity): 688.2 (100). Calcd for C_38_H_49_CuN_4_O_4_ ([M − H]^−^): 688.30. HRMS (ESI-TOF): *m*/*z* 688.3056. Calcd for C_36_H_49_CuN_4_O_4_ ([M − H]^−^]): 688.3055. This compound was further characterized by the X-ray diffraction analysis.

#### 3.1.7. Cu^II^ Complex of 1,3-di(5-phenylpyrrol-2-yl)-1,3-propanedione (2a)

A mixture of 1,3-di(5-phenylpyrrol-2-yl)-1,3-propanedione **2a′ [21]** (30.0 mg, 0.075 mmol) and Cu(OAc)_2_ (6.4 mg, 0.035 mmol) in CH_2_Cl_2_ (20 mL) and MeOH (30 mL) was stirred at 25 °C for 0.5 h. The precipitate was collected and washed with CH_2_Cl_2_ to afford **2a** (20.2 mg, 0.026 mmol, 69%) as a yellow solid. MALDI-TOF-MS: *m*/*z* (% intensity): 768.2 (100). Calcd for C_46_H_33_CuN_4_O_4_ ([M − H]^−^): 768.18. HRMS (ESI-TOF): *m*/*z* 768.1803. Calcd for C_46_H_33_CuN_4_O_4_ ([M − H]^−^]): 768.1803. This compound was further characterized by the X-ray diffraction analysis. The UV/vis absorption spectrum was not measured due to the low solubility.

#### 3.1.8. Cu^II^ complex of 1,3-di(5-(2-methoxyphenyl)pyrrol-2-yl)-1,3-propanedione (2b)

A mixture of 1,3-di(5-(2-methoxyphenyl)pyrrol-2-yl)-1,3-propanedione **2b′** [22] (21.3 mg, 0.051 mmol) and Cu(OAc)_2_ (4.65 mg, 0.026 mmol) in CH_2_Cl_2_ (20 mL) and MeOH (20 mL) was stirred at 25 °C for 0.5 h. After the solvent was evaporated, the residue was recrystallized from MeOH/*n*-hexane to afford **2b** (14.5 mg, 0.016 mmol, 63%) as a brown solid. UV/vis (CH_2_Cl_2_, λ_max_[nm] (ε, 10^4^ M^−1^cm^−1^)): 454 (9.3). MALDI-TOF-MS: *m/z* (% intensity): 888.2 (100). Calcd for C_50_H_41_CuN_4_O_8_ ([M − H]^−^): 888.22. HRMS (ESI-TOF): *m*/*z* 888.2226. Calcd for C_50_H_41_CuN_4_O_8_ ([M − H]^−^]): 888.2226. This compound was further characterized by the X-ray diffraction analysis.

#### 3.1.9. Cu^II^ complex of 1,3-di(5-(3-methoxyphenyl)pyrrol-2-yl)-1,3-propanedione (2c)

A mixture of 1,3-di(5-(3-methoxyphenyl)pyrrol-2-yl)-1,3-propanedione **2c′ [22]** (35.5 mg, 0.086 mmol) and Cu(OAc)_2_ (7.76 mg, 0.043 mmol) in CH_2_Cl_2_ (30 mL) and MeOH (30 mL) was stirred at 25 °C for 0.5 h. After the solvent was evaporated, the residue was recrystallized from MeOH/*n*-hexane to afford **2c** (30.5 mg, 0.034 mmol, 80%) as a brown solid. UV/vis (THF, λ_max_[nm] (ε, 10^4^ M^−1^cm^−1^)): 430 (8.2). MALDI-TOF-MS: *m/z* (% intensity): 888.3 (100). Calcd for C_50_H_41_CuN_4_O_8_ ([M − H]^−^): 888.22. HRMS (ESI-TOF): *m*/*z* 888.2224. Calcd for C_50_H_41_CuN_4_O_8_ ([M − H]^−^]): 888.2226. This compound was further characterized by the X-ray diffraction analysis.

#### 3.1.10. Cu^II^ complex of 1,3-di(5-(4-methoxyphenyl)pyrrol-2-yl)-1,3-propanedione (2d)

A mixture of 1,3-di(5-(4-methoxyphenyl)pyrrol-2-yl)-1,3-propanedione **2d′ [22]** (22.5 mg, 0.054 mmol) and Cu(OAc)_2_ (4.91 mg, 0.027 mmol) in CH_2_Cl_2_ (20 mL) and MeOH (20 mL) was stirred at 25 °C for 0.5 h. The precipitate was collected and washed with CH_2_Cl_2_ to afford **2d** (16.8 mg, 0.019 mmol, 72%) as a yellow solid. MALDI-TOF-MS: *m*/*z* (% intensity): 888.2 (100). Calcd for C_50_H_41_CuN_4_O_8_ ([M − H]^−^): 888.22. HRMS (ESI-TOF): *m*/*z* 888.2225. Calcd for C_50_H_41_CuN_4_O_8_ ([M − H]^−^]): 888.2226. This compound was further characterized by the X-ray diffraction analysis. The UV/vis absorption spectrum was not measured due to the low solubility.

#### 3.1.11. Cu^II^ complex of 1,3-bis(5-(3,4-dimethoxyphenyl)pyrrol-2-yl)-1,3-propanedione (2e)

A mixture of **2e′** (31.0 mg, 0.065 mmol) and Cu(OAc)_2_ (5.72 mg, 0.033 mmol) in CH_2_Cl_2_ (20 mL) and MeOH (40 mL) was stirred at 25 °C for 0.5 h. The precipitate was collected and washed with CH_2_Cl_2_ to afford **2e** (32.0 mg, 0.032 mmol, 98%) as a yellow solid. UV/vis (CH_2_Cl_2_, λ_max_[nm] (ε, 10^4^ M^−1^cm^−1^)): 444 (9.1). MALDI-TOF-MS: *m/z* (% intensity): 1008.4 (100). Calcd for C_54_H_49_CuN_4_O_12_ ([M − H]^−^): 1008.26. HRMS (ESI-TOF): *m*/*z* 1008.2649. Calcd for C_54_H_49_CuN_4_O_12_ ([M − H]^−^]): 1008.2648. This compound was further characterized by the X-ray diffraction analysis.

#### 3.1.12. Cu^II^ complex of 1,3-bis(5-(3,4,5-trimethoxyphenyl)pyrrol-2-yl)-1,3-propanedione (2f)

A mixture of 1,3-bis(5-(3,4,5-trimethoxyphenyl)pyrrol-2-yl)-1,3-propanedione **2f′** [21] (40.0 mg, 0.075 mmol) and Cu(OAc)_2_ (6.4 mg, 0.035 mmol) in CH_2_Cl_2_ (20 mL) and MeOH (30 mL) was stirred at 25 °C for 0.5 h. The precipitate was collected and washed with CH_2_Cl_2_ to afford **2f** (36.14 mg, 0.032 mmol, 91%) as a brown solid. UV/vis (THF, λ_max_[nm] (ε, 10^4^ M^−1^cm^−1^)): 448 (9.5). MALDI-TOF-MS: *m*/*z* (% intensity): 1128.3 (100). Calcd for C_58_H_57_CuN_4_O_16_ ([M − H]^−^): 1128.31. HRMS (ESI-TOF): *m*/*z* 1128.3071. Calcd for C_58_H_57_CuN_4_O_16_ ([M − H]^−^]): 1128.3071. This compound was further characterized by the X-ray diffraction analysis.

#### 3.1.13. Cu^II^ complex of 1,3-bis(5-(3,4-dihexadecyloxyphenyl)pyrrol-2-yl)-1,3-propanedione (3a)

A mixture of **3a′** (99.56 mg, 0.076 mmol) and Cu(OAc)_2_ (6.87 mg, 0.038 mmol) in CHCl_3_ (30 mL) was stirred at 25 °C for 0.5 h. After the solvent was evaporated, the residue was reprecipitated from CHCl_3_/MeOH to afford **3a** (94.28 mg, 0.035 mmol, 93%) as a brown solid. UV/vis (CH_2_Cl_2_, λ_max_[nm] (ε, 10^4^ M^−1^cm^−1^)): 447 (8.6). MALDI-TOF-MS: *m/z* (% intensity): 2692.2 (100). Calcd for C_174_H_289_CuN_4_O_12_ ([M − H]^−^): 2692.15.

#### 3.1.14. Cu^II^ complex of 1,3-bis(5-(3,4,5-trihexadecyloxyphenyl)pyrrol-2-yl)-1,3-propanedione (3b)

A mixture of 1,3-bis(5-(3,4,5-trihexadecyloxyphenyl)pyrrol-2-yl)-1,3-propanedione **3b′ [21]** (150.0 mg, 0.084 mmol) and Cu(OAc)_2_ (7.58 mg, 0.042 mmol) in MeOH (40 mL) was stirred at 25 °C for 0.5 h. After the solvent was evaporated, the residue was reprecipitated from CHCl_3_/MeOH to afford **2h** (138.1 mg, 0.038 mmol, 90%) as a brown solid. UV/vis (CH_2_Cl_2_, λ_max_[nm] (ε, 10^4^ M^−1^cm^−1^)): 448 (8.3). MALDI-TOF-MS: *m/z* (% intensity): 3654.1 (100). Calcd for C_238_H_417_CuN_4_O_16_ ([M − H]^−^): 3654.13.

### 3.2. Method for Single-Crystal X-ray Analysis

Crystallographic data are summarized in the Supplementary Materials. A single crystal of **1a** was obtained by vapor diffusion of *n*-hexane into a MeOH solution of **1a**. The data crystal was a yellow prism of approximate dimensions 0.30 × 0.10 × 0.05 mm. A single crystal of **1b** was obtained by vapor diffusion of *n*-hexane into a CHCl_3_ solution of **1b**. The data crystal was a violet prism of approximate dimensions 0.50 × 0.30 × 0.20 mm. A single crystal of **2a** was obtained by vapor diffusion of water into an *N*-methyl-2-pyrrolidone and MeOH mixed solution of **2a**. The data crystal was a yellow prism of approximate dimensions 0.10 × 0.10 × 0.10 mm. A single crystal of **2b** was obtained by vapor diffusion of *n*-hexane into a MeOH solution of **2b**. The data crystal was a yellow prism of approximate dimensions 0.20 × 0.15 × 0.10 mm. A single crystal of **2c** was obtained by vapor diffusion of *n*-hexane into a MeOH solution of **2c**. The data crystal was an orange prism of approximate dimensions 0.60 × 0.40 × 0.20 mm. A single crystal of **2f** was obtained by vapor diffusion of *n*-hexane into a mixture of MeOH and CH_2_Cl_2_ solution of **2f**. The data crystal was an orange prism of approximate dimensions 0.40 × 0.20 × 0.10 mm. The data of **1a**,**b** and **2a**–**c**,**f** were collected at 123 K on a Rigaku RAXIS-RAPID diffractometer (Rigaku Corp., Tokyo, Japan) with graphite monochromated Mo-Kα (λ = 0.71075 Å). All the structures were solved by the dual-space method. The structures were refined by a full-matrix least-squares method using a SHELXL 2014 [34] (Yadokari-XG) [35,36]. In each structure, the non-hydrogen atoms were refined anisotropically. All the hydrogen atom positions were placed at calculated positions and rode on the atom of attachment except for the hydrogen atoms of water in **1a**. For **2c**,**f**, the disordered solvents, presumably *N*-methylpyrrolidone and CH_2_Cl_2_, respectively, were removed using the SQUEEZE protocol included in PLATON [37]. CIF files (CCDC-2051817–2051822) can be obtained free of charge from the Cambridge Crystallographic Data Centre.

### 3.3. DFT Caluculations

DFT calculations for the geometrical optimizations were carried out using the *Gaussian 09* program [23].

### 3.4. Differential Scanning Calorimetry (DSC)

The phase transitions were measured on a differential scanning calorimetry (Shimadzu DSC-60 (Shimadzu Corp., Kyoto, Japan)).

### 3.5. Polarizing Optical Microscopy (POM)

The POM observation was carried out with a Nikon ECLIPSE E600POL polarizing optical microscope (Nikon Corp., Tokyo, Japan) equipped with a Mettler Toledo FP-82 HT hot stage system (Mettler Toledo, Columbus, OH, USA).

### 3.6. Synchrotron X-ray Diffraction Analysis (XRD)

High-resolution XRD analyses were carried out using a synchrotron radiation X-ray beam with a wavelength of 1.00 Å on BL40B2 at SPring-8 (Hyogo, Japan). The diffractions were detected by a large Debye-Scherrer camera with an imaging plate. The camera lengths were set at 550.5 mm. The diffraction patterns were obtained with a 0.01° step in 2θ. An exposure time of the X-ray beam was 10 s.

## 4. Conclusions

Cu^II^ complexes of dipyrrolyldiketones exhibited planar geometries based on their square planar coordination. Rectangular structures were formed upon extending the long axis of these molecules with the introduction of aryl units at the pyrrole α-positions. Planar dipyrrolyldiketone Cu^II^ complexes were suitable for the generation of stacking-based molecular assemblies, as observed in single-crystal structures. Furthermore, the introduction of the aliphatic alkoxy chains induced mesophases as dimension-controlled assemblies based on the characteristic geometries of the formation of dipyrrolyldiketone Cu^II^ complexes. The molecular design aimed at tuning the conformation of 1,3-diketone metal complexes developed in this study might enable further modifications of π-electronic systems for providing electronically and optically attractive materials.

## Data Availability

The data presented in this study are available in the article and supplementary materials.

## Supplementary Materials

The following are available online, Figure S1: Synthetic scheme; Figures S2–S5: ^1^H NMR of **2d′**, **3a′**, and precursors of **3a′**; Figure S6: UV/vis absorption spectrum of **1a**; Figures S7–S12: Ortep drawings of **1a**,**b** and **2a**–**c**,**f**; Figures S13–S18: Packing diagrams for crystal structures; Figure S19: Hirshfeld surfaces of **2b**,**c**; Figure S20: Optimized structure of **1a**; Figure S21: Molecular orbitals of **1a**; Figure S22: TD-DFT calculation of **1a**; Figure S23: DSC thermograms of **3a**,**b**; Figure S24: POM textures of **3a**,**b**; Figures S25 and S27: XRD patterns of **3a**,**b**; Figures S26 and S28: Possible packing models of **3a**,**b**; Table S1: Crystallographic details; Tables S2 and S3: Summary of XRD data.

## Sample Availability

Samples of the compounds are available from the authors.

## Abbreviations

NMR	Nuclear magnetic resonance
MALDI-TOF-MS	Matrix-assisted laser desorption/ionization time-of-flight mass spectrometry
ESI-TOF-MS	Electrospray ionization time-of-flight mass spectrometry
DFT	Density functional theory
DSC	Differential scanning calorimetry
POM	Polarized optical microscopy
XRD	X-ray diffraction
TLC	Thin-layer chromatography

## References

[1] Koch N. (2015). Supramolecular Materials for Opto-Electronics.

[2] Haketa Y., Maeda H. (2018). Dimension-Controlled π-Electronic Ion-Pairing Assemblies. Bull. Chem. Soc. Jpn..

[3] Haketa Y., Urakawa K., Maeda H. (2020). First decade of π-electronic ion-pairing assemblies. Mol. Syst. Des. Eng..

[4] Haketa Y., Sasaki S., Ohta N., Masunaga H., Ogawa H., Mizuno N., Araoka F., Takezoe H., Maeda H. (2010). Oriented salts: Dimension-controlled charge-by-charge assemblies from planar receptor–anion complexes. Angew. Chem. Int. Ed..

[5] Maeda H., Naritani K., Honsho Y., Seki S. (2011). Anion modules: Building blocks of supramolecular assemblies by combination with π-conjugated anion receptors. J. Am. Chem. Soc..

[6] Haketa Y., Honsho Y., Seki S., Maeda H. (2012). Ion materials comprising planar charged species. Chem. Eur. J..

[7] Dong B., Sakurai T., Honsho Y., Seki S., Maeda H. (2013). Cation modules as building blocks forming supramolecular assemblies with planar receptor-anion complexes. J. Am. Chem. Soc..

[8] Dong B., Sakurai T., Bando Y., Seki S., Takaishi K., Uchiyama M., Muranaka A., Maeda H. (2013). Ion-based materials derived from positively and negatively charged chloride complexes of π-conjugated molecules. J. Am. Chem. Soc..

[9] Vigato P.A., Peruzzo V., Tamburini S. (2009). The evolution of β-diketophenol ligands and related complexes. Coord. Chem. Rev..

[10] Brock A.J., Clegg J.K., Li F., Lindoy L.F. (2018). Recent developments in the metallo-supramolecular chemistry of oligo-β-diketonato ligands. Coord. Chem. Rev..

[11] Zheng H., Lai C.K., Swager T.M. (1995). Controlling intermolecular interactions between metallomesogens: Side-chain effects in discotic copper, palladium, and vanadyl bis(β-diketonates. Chem. Mater..

[12] Ohta K., Yokoyama M., Kusubayashi S., Mikawa H. (1980). Square-planar *trans*-Bis-(1-*p*-n-octylphenylbutane-1,3-dionato)copper(II), a new compound exhibiting three kinds of ‘double melting′ behaviour. J. Chem. Soc. Chem. Commun..

[13] Ohta K., Ishii A., Yamamoto I., Matsuzaki K. (1984). Discotic liquid crystals of organocopper complexes: The substituent effects. J. Chem. Soc. Chem. Commun..

[14] Thompson N.J., Goodby J.W., Toyne K.J. (1992). Liquid-crystalline polymesomorphism in copper(II) complexes of β-diketones. Mol. Cryst. Liq. Cryst..

[15] Poelsma S.N., Servante A.H., Fanizzi F.P., Maitlis P.M. (1994). Discotic metallomesogens: Synthesis and properties of square planar metal bis(β-diketonate) complexes. Liq. Cryst..

[16] Prasad V., Sadashiva B.K. (1995). Liquid crystalline behaviour in some homologous series of β-diketones and a few of their copper(II) and palladium(II) complexes. Mol. Cryst. Liq. Cryst..

[17] Schmidt A., Heinrich B., Kirscher G., Chaumont A., Henry M., Kyritsakas N., Haketa Y., Maeda H., Mobian P. (2020). Dipyrrolyldiketonato Titanium(IV) complexes from monomeric to multinuclear architectures: Synthesis, stability and liquid crystal properties. Inorg. Chem..

[B18-molecules-26-00861] 18.Pt^II^ complexes of dipyrrolyldiketones have also been prepared and will be reported elsewhere.

[19] Maeda H., Terasaki M., Haketa Y., Mihashi Y., Kusunose Y. (2008). BF_2_ complexes of α-alkyl-substituted dipyrrolyldiketones as acyclic anion receptors. Org. Biomol. Chem..

[20] Maeda H., Kusunose Y., Mihashi Y., Mizoguchi T. (2007). BF_2_ Complexes of β-tetraethyl-substituted dipyrrolyldiketones as anion receptors: Potential building subunits for oligomeric systems. J. Org. Chem..

[21] Maeda H., Haketa Y., Nakanishi T. (2007). Aryl-substituted C_3_-bridged oligopyrroles as anion receptors for formation of supramolecular organogels. J. Am. Chem. Soc..

[22] Maeda H., Eifuku N. (2009). Alkoxy-substituted derivatives of π-conjugated acyclic anion receptors: Effects of substituted positions. Chem. Lett..

[23] Frisch M.J., Trucks G.W., Schlegel H.B., Scuseria G.E., Robb M.A., Cheeseman J.R., Scalmani G., Barone V., Mennucci B., Petersson G.A. (2013). Gaussian 09.

[24] Yang L., Powell D.R., Houser R.P. (2007). Structural variation in copper(I) complexes with pyridylmethylamide ligands: Structural analysis with a new four-coordinate geometry index, τ_4_. Dalton Trans..

[25] Turner M.J., McKinnon J.J., Wolff S.K., Grimwood D.J., Spackman P.R., Jayatilaka D., Spackman M.A. (2017). CrystalExplorer17.

[26] McKinnon J.J., Spackman M.A., Mitchell A.S. (2004). Novel tools for visualizing and exploring intermolecular interactions in molecular crystals. Acta Crystallogr. Sect. B.

[27] Tan S.L., Jotani M.M., Tiekink E.R.T. (2019). Utilizing Hirshfeld surface calculations, non-covalent interaction (NCI) plots and the calculation of interaction energies in the analysis of molecular packing. Acta Crystallogr. Sect. E.

[B28-molecules-26-00861] 28.Similar characteristic Hirshfeld surfaces were observed in the crystal-state π–π stacking structures of large aromatic hydrocarbons such as hexabenzocoronene: See also [26].

[29] Tiekink E.R.T., Zukerman-Schpector J. (2012). The importance of Pi-Interactions in Crystal Engineering: Frontiers in Crystal Engineering.

[30] For the elucidation of the mesophase structures: Ohta, K (2020). Physics and Chemistry of Molecular Assemblies.

[31] Belov D.S., Ivanov V.N., Curreli F., Kurkin A.V., Altieri A., Debnath A.K. (2017). Synthesis of 5-arylpyrrole-2-carboxylic acids as key intermediates for nbd series hiv-1-entry inhibitors. Synthesis.

[32] Herod J.D., Bates M.A., Whitwood A.C., Bruce D.W. (2019). Ionic *N*-phenylpyridinium tetracatenar mesogens: Competing driving forces in mesophase formation and unprecedented difference in phase stabilization within a homologous series. Soft Matter.

[33] Maeda H., Kusunose Y., Terasaki M., Ito Y., Fujimoto C., Fujii R., Nakanishi T. (2007). Micro- and nanometer-scale porous, fibrous, and sheet architectures constructed by supramolecular assemblies of dipyrrolyldiketones. Chem. Asian J..

[34] Sheldrick G.M. (2008). Crystal structure refinement with *SHELXL*. Acta Crystallogr. Sect. A.

[35] (2001). Yadokari-XG.

[36] Kabuto C., Akine S., Nemoto T., Kwon E. (2009). Release of software (Yadokari-XG 2009) for crystal structure analyses. J. Cryst. Soc. Jpn..

[37] Spek A.L. (2009). Structure validation in chemical crystallography. Acta Crystallogr. Sect. D.

