# Supplementary material for: Supramolecular Assemblies of Dipyrrolyldiketone CuII Complexes
Source: Molecules. 2021 Feb 6;26(4):861. doi: 10.3390/molecules26040861 (PMC7914842; doi:10.3390/molecules26040861)

checkCIF/PLATON report

Structure factors have been supplied for datablock(s) 70316mae_yh

THIS REPORT IS FOR GUIDANCE ONLY. IF USED AS PART OF A REVIEW PROCEDURE FOR PUBLICATION, IT SHOULD NOT REPLACE THE EXPERTISE OF AN EXPERIENCED CRYSTALLOGRAPHIC REFEREE.

No syntax errors found. CIF dictionary Interpreting this report

Datablock: 70316mae_yh

```
Bond precision:  C-C = 0.0043 A          Wavelength=0.71075

Cell:           a=27.682(12)    b=5.901(3)          c=16.000(8)
                alpha=90        beta=107.928(16)    gamma=90
Temperature:    123 K

                Calculated                 Reported
Volume          2487(2)                    2487(2)
Space group     C 2/c                      C 2/c
Hall group      -C 2yc                     -C 2yc
Moiety formula  C26 H26 Cu N4 O4, 2(H2 O)  C26 H26 Cu N4 O4, 2(H2 O)
Sum formula     C26 H30 Cu N4 O6           C26 H30 Cu N4 O6
Mr              558.09                     558.08
Dx,g cm-3       1.490                      1.491
Z               4                          4
Mu (mm-1)       0.928                      0.928
F000            1164.0                     1164.0
F000'           1165.74
h,k,lmax        35,7,20                    35,7,20
Nref            2858                       2846
Tmin,Tmax       0.895,0.955
Tmin'           0.757

Correction method= Not given

Data completeness= 0.996          Theta(max)= 27.472

R(reflections)= 0.0495( 2007)     wR2(reflections)= 0.1003( 2846)

S = 1.064                   Npar= 179
```

The following ALERTS were generated. Each ALERT has the format
test-name_ALERT_alert-type_alert-level.
Click on the hyperlinks for more details of the test.

Alert level B

```
PLAT420_ALERT_2_B D-H Without Acceptor       O3       --H3B      .     Please Check
```

Alert level C

```
PLAT417_ALERT_2_C Short Inter D-H..H-D       H1C      ..H3A      .       2.14 Ang.
                                                1-x,-y,1-z  =      5_656 Check
PLAT906_ALERT_3_C Large K Value in the Analysis of Variance ......      3.147 Check
```

Alert level G

```
PLAT007_ALERT_5_G Number of Unrefined Donor-H Atoms ..............          2 Report
PLAT128_ALERT_4_G Alternate Setting for Input Space Group     C2/c       I2/a Note
PLAT883_ALERT_1_G No Info/Value for _atom_sites_solution_primary .     Please Do !
PLAT910_ALERT_3_G Missing # of FCF Reflection(s) Below Theta(Min).          3 Note
PLAT912_ALERT_4_G Missing # of FCF Reflections Above STh/L=  0.600          8 Note
PLAT941_ALERT_3_G Average HKL Measurement Multiplicity ...........        4.0 Low
PLAT965_ALERT_2_G The SHELXL WEIGHT Optimisation has not Converged     Please Check
PLAT978_ALERT_2_G Number C-C Bonds with Positive Residual Density.          4 Info
```

---

0 **ALERT level A** = Most likely a serious problem - resolve or explain
1 **ALERT level B** = A potentially serious problem, consider carefully
2 **ALERT level C** = Check. Ensure it is not caused by an omission or oversight
8 **ALERT level G** = General information/check it is not something unexpected

1 ALERT type 1 CIF construction/syntax error, inconsistent or missing data
4 ALERT type 2 Indicator that the structure model may be wrong or deficient
3 ALERT type 3 Indicator that the structure quality may be low
2 ALERT type 4 Improvement, methodology, query or suggestion
1 ALERT type 5 Informative message, check

Validation response form

Please find below a validation response form (VRF) that can be filled in and pasted into your CIF.

```
# start Validation Reply Form
_vrf_PLAT420_70316mae_yh
;
PROBLEM: D-H Without Acceptor       O3       --H3B      .     Please Check
RESPONSE: ...
;
_vrf_PLAT417_70316mae_yh
;
PROBLEM: Short Inter D-H..H-D       H1C      ..H3A      .       2.14 Ang.
RESPONSE: ...
;
_vrf_PLAT906_70316mae_yh
;
PROBLEM: Large K Value in the Analysis of Variance ......      3.147 Check
RESPONSE: ...
;
# end Validation Reply Form
```

It is advisable to attempt to resolve as many as possible of the alerts in all categories. Often the minor alerts point to easily fixed oversights, errors and omissions in your CIF or refinement strategy, so attention to these fine details can be worthwhile. In order to resolve some of the more serious problems it may be necessary to carry out additional measurements or structure refinements. However, the purpose of your study may justify the reported deviations and the more serious of these should normally be commented upon in the discussion or experimental section of a paper or in the "special_details" fields of the CIF. checkCIF was carefully designed to identify outliers and unusual parameters, but every test has its limitations and alerts that are not important in a particular case may appear. Conversely, the absence of alerts does not guarantee there are no aspects of the results needing attention. It is up to the individual to critically assess their own results and, if necessary, seek expert advice.

Publication of your CIF in IUCr journals

A basic structural check has been run on your CIF. These basic checks will be run on all CIFs submitted for publication in IUCr journals (*Acta Crystallographica*, *Journal of Applied Crystallography*, *Journal of Synchrotron Radiation*); however, if you intend to submit to *Acta Crystallographica Section C* or *E* or *IUCrData*, you should make sure that full publication checks are run on the final version of your CIF prior to submission.

Publication of your CIF in other journals

Please refer to the *Notes for Authors* of the relevant journal for any special instructions relating to CIF submission.

PLATON version of 05/12/2020; check.def file version of 05/12/2020

Datablock 70316mae_yh - ellipsoid plot

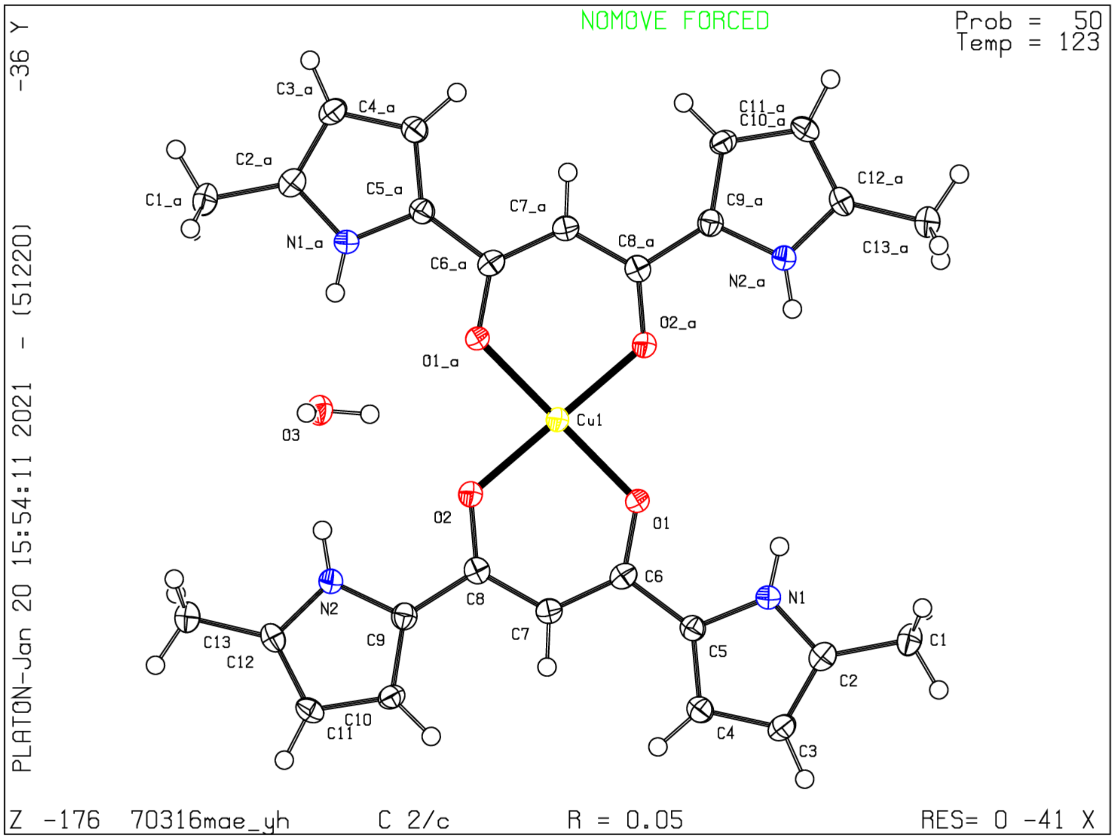

checkCIF/PLATON report

Structure factors have been supplied for datablock(s) 70315mae_a_yh

THIS REPORT IS FOR GUIDANCE ONLY. IF USED AS PART OF A REVIEW PROCEDURE FOR PUBLICATION, IT SHOULD NOT REPLACE THE EXPERTISE OF AN EXPERIENCED CRYSTALLOGRAPHIC REFEREE.

No syntax errors found.   CIF dictionary   Interpreting this report

Datablock: 70315mae_a_yh

Bond precision: C-C = 0.0044 A   Wavelength=0.71075

Cell:	a=14.579(5)	b=25.909(6)	c=11.798(3)
	alpha=90	beta=90	gamma=90
Temperature:	123 K		

	Calculated	Reported
Volume	4456(2)	4457(2)
Space group	P c c n	P c c n
Hall group	-P 2ab 2ac	-P 2ab 2ac
Moiety formula	C38 H50 Cu N4 O4, 2(C H Cl3)	C38 H50 Cu N4 O4, 2(C H Cl3)
Sum formula	C40 H52 Cl6 Cu N4 O4	C40 H52 Cl6 Cu N4 O4
Mr	929.11	929.09
Dx,g cm-3	1.385	1.385
Z	4	4
Mu (mm-1)	0.893	0.893
F000	1932.0	1932.0
F000’	1937.38	
h,k,lmax	18,33,15	18,33,15
Nref	5112	5014
Tmin,Tmax	0.732,0.836	
Tmin’	0.634	

Correction method= Not given

Data completeness= 0.981   Theta(max)= 27.485

R(reflections)= 0.0507(3587)   wR2(reflections)= 0.1223(5014)

S = 1.026   Npar= 303

The following ALERTS were generated. Each ALERT has the format
test-name_ALERT_alert-type_alert-level.
Click on the hyperlinks for more details of the test.

Alert level C

```
PLAT057_ALERT_3_C Correction for Absorption Required   RT(exp) ...       1.14 Do !
PLAT220_ALERT_2_C NonSolvent   Resd 1  C   Ueq(max)/Ueq(min) Range        4.2 Ratio
PLAT222_ALERT_3_C NonSolvent Resd 1  H   Uiso(max)/Uiso(min) Range        4.7 Ratio
PLAT906_ALERT_3_C Large K Value in the Analysis of Variance ......      4.425 Check
PLAT910_ALERT_3_C Missing # of FCF Reflection(s) Below Theta(Min).         6 Note
PLAT911_ALERT_3_C Missing FCF Refl Between Thmin & STh/L=    0.600        36 Report
```

Alert level G

```
PLAT007_ALERT_5_G Number of Unrefined Donor-H Atoms .............          2 Report
PLAT301_ALERT_3_G Main Residue  Disorder .............(Resd  1  )        9% Note
PLAT302_ALERT_4_G Anion/Solvent/Minor-Residue Disorder (Resd  2  )       75% Note
PLAT883_ALERT_1_G No Info/Value for _atom_sites_solution_primary .     Please Do !
PLAT912_ALERT_4_G Missing # of FCF Reflections Above STh/L=  0.600        57 Note
PLAT965_ALERT_2_G The SHELXL WEIGHT Optimisation has not Converged     Please Check
PLAT978_ALERT_2_G Number C-C Bonds with Positive Residual Density.         4 Info
```

```
   0 ALERT level A = Most likely a serious problem - resolve or explain
   0 ALERT level B = A potentially serious problem, consider carefully
   6 ALERT level C = Check. Ensure it is not caused by an omission or oversight
   7 ALERT level G = General information/check it is not something unexpected

   1 ALERT type 1 CIF construction/syntax error, inconsistent or missing data
   3 ALERT type 2 Indicator that the structure model may be wrong or deficient
   6 ALERT type 3 Indicator that the structure quality may be low
   2 ALERT type 4 Improvement, methodology, query or suggestion
   1 ALERT type 5 Informative message, check
```

Validation response form

Please find below a validation response form (VRF) that can be filled in and pasted into your CIF.

```
# start Validation Reply Form
_vrf_PLAT057_70315mae_a_yh
;
PROBLEM: Correction for Absorption Required   RT(exp) ...       1.14 Do !
RESPONSE: ...
;
_vrf_PLAT220_70315mae_a_yh
;
PROBLEM: NonSolvent   Resd 1  C   Ueq(max)/Ueq(min) Range        4.2 Ratio
RESPONSE: ...
;
_vrf_PLAT222_70315mae_a_yh
;
PROBLEM: NonSolvent Resd 1  H   Uiso(max)/Uiso(min) Range        4.7 Ratio
RESPONSE: ...
;
_vrf_PLAT906_70315mae_a_yh
;
PROBLEM: Large K Value in the Analysis of Variance ......      4.425 Check
RESPONSE: ...
;
_vrf_PLAT910_70315mae_a_yh
;
PROBLEM: Missing # of FCF Reflection(s) Below Theta(Min).         6 Note
RESPONSE: ...
;
```

```
_vrf_PLAT911_70315mae_a_yh
;
PROBLEM: Missing FCF Refl Between Thmin & STh/L=    0.600         36 Report
RESPONSE: ...
;
# end Validation Reply Form
```

It is advisable to attempt to resolve as many as possible of the alerts in all categories. Often the minor alerts point to easily fixed oversights, errors and omissions in your CIF or refinement strategy, so attention to these fine details can be worthwhile. In order to resolve some of the more serious problems it may be necessary to carry out additional measurements or structure refinements. However, the purpose of your study may justify the reported deviations and the more serious of these should normally be commented upon in the discussion or experimental section of a paper or in the "special_details" fields of the CIF. checkCIF was carefully designed to identify outliers and unusual parameters, but every test has its limitations and alerts that are not important in a particular case may appear. Conversely, the absence of alerts does not guarantee there are no aspects of the results needing attention. It is up to the individual to critically assess their own results and, if necessary, seek expert advice.

Publication of your CIF in IUCr journals

A basic structural check has been run on your CIF. These basic checks will be run on all CIFs submitted for publication in IUCr journals (*Acta Crystallographica*, *Journal of Applied Crystallography*, *Journal of Synchrotron Radiation*); however, if you intend to submit to *Acta Crystallographica Section C* or *E* or *IUCrData*, you should make sure that full publication checks are run on the final version of your CIF prior to submission.

Publication of your CIF in other journals

Please refer to the *Notes for Authors* of the relevant journal for any special instructions relating to CIF submission.

PLATON version of 05/12/2020; check.def file version of 05/12/2020

Datablock 70315mae_a_yh - ellipsoid plot

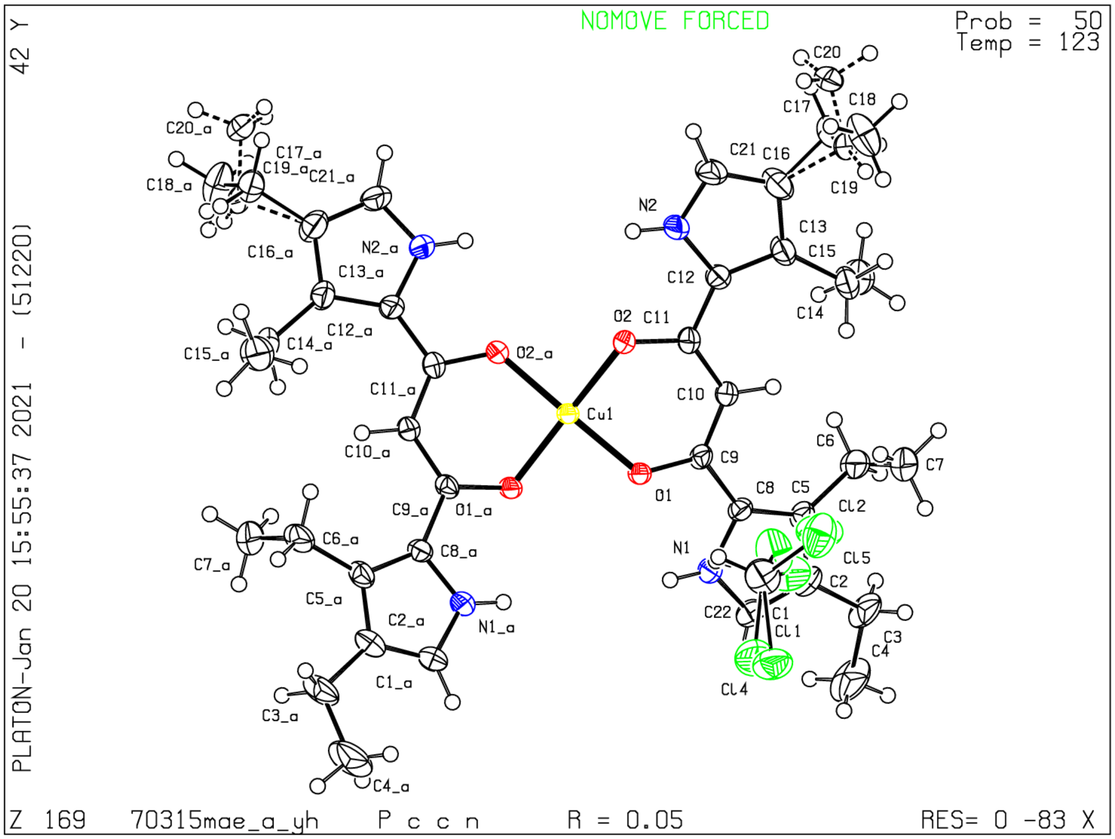

checkCIF/PLATON report

Structure factors have been supplied for datablock(s) aPhCu3

THIS REPORT IS FOR GUIDANCE ONLY. IF USED AS PART OF A REVIEW PROCEDURE FOR PUBLICATION, IT SHOULD NOT REPLACE THE EXPERTISE OF AN EXPERIENCED CRYSTALLOGRAPHIC REFEREE.

No syntax errors found. CIF dictionary Interpreting this report

Datablock: aPhCu3

```
Bond precision:  C-C = 0.0141 A          Wavelength=0.71075

Cell:           a=9.08(2)          b=16.06(4)        c=16.68(4)
                alpha=114.83(7)    beta=96.05(11)    gamma=100.27(7)
Temperature:    123 K
```

	Calculated	Reported
Volume	2127(9)	2129(9)
Space group	P -1	P -1
Hall group	-P 1	-P 1
Moiety formula	C46 H34 Cu N4 O4, C5 H9 N O, 2(C H4 O)	C46 H34 Cu N4 O4, C5 H9 N O, 2(C H4 O)
Sum formula	C53 H51 Cu N5 O7	C53 H51 Cu N5 O7
Mr	933.54	933.52
Dx,g cm-3	1.458	1.456
Z	2	2
Mu (mm-1)	0.578	0.577
F000	978.0	978.0
F000’	979.02	
h,k,lmax	11,20,21	11,20,21
Nref	9763	9022
Tmin,Tmax	0.944,0.944	0.945,0.945
Tmin’	0.944	

```
Correction method= # Reported T Limits: Tmin=0.945 Tmax=0.945
AbsCorr = NONE

Data completeness= 0.924          Theta(max)= 27.485

R(reflections)= 0.1210( 3484)     wR2(reflections)= 0.3721( 9022)

S = 0.981                  Npar= 592
```

The following ALERTS were generated. Each ALERT has the format
test-name_ALERT_alert-type_alert-level.
Click on the hyperlinks for more details of the test.

Alert level A

```
PLAT029_ALERT_3_A _diffrn_measured_fraction_theta_full value Low .      0.935 Why?
```

Author Response: Some reflections were not measured due to the problems derived from the data collecting strategy for the crystal measurement.

Alert level B

```
PLAT026_ALERT_3_B Ratio Observed / Unique Reflections (too) Low ..        39% Check
PLAT084_ALERT_3_B High wR2 Value (i.e. > 0.25) ...................       0.37 Report
PLAT417_ALERT_2_B Short Inter D-H..H-D       H1A      ..H68       .       2.06 Ang.
                                                      x,y,z  =      1_555 Check
PLAT417_ALERT_2_B Short Inter D-H..H-D       H4A      ..H6        .       1.83 Ang.
                                                2-x,1-y,1-z  =      2_766 Check
PLAT910_ALERT_3_B Missing # of FCF Reflection(s) Below Theta(Min).         17 Note
```

Alert level C

```
RINTA01_ALERT_3_C  The value of Rint is greater than 0.12
            Rint given   0.157
PLAT020_ALERT_3_C The Value of Rint is Greater Than 0.12 .........      0.157 Report
PLAT082_ALERT_2_C High R1 Value ..................................       0.12 Report
PLAT148_ALERT_3_C s.u. on the      a    - Axis is (Too) Large ....      0.020 Ang.
PLAT148_ALERT_3_C s.u. on the      b    - Axis is (Too) Large ....      0.040 Ang.
PLAT148_ALERT_3_C s.u. on the      c    - Axis is (Too) Large ....      0.040 Ang.
PLAT149_ALERT_3_C s.u. on the   beta    Angle is Too Large .......       0.11 Degree
PLAT202_ALERT_3_C Isotropic non-H Atoms in Anion/Solvent .........          1 Check
              O5
PLAT234_ALERT_4_C Large Hirshfeld Difference C3       --C4        .       0.20 Ang.
PLAT331_ALERT_2_C Small Aver Phenyl C-C Dist C18      --C23       .       1.37 Ang.
PLAT331_ALERT_2_C Small Aver Phenyl C-C Dist C24      --C29       .       1.37 Ang.
PLAT331_ALERT_2_C Small Aver Phenyl C-C Dist C41      --C46       .       1.37 Ang.
PLAT341_ALERT_3_C Low Bond Precision on  C-C Bonds ...............    0.01412 Ang.
PLAT417_ALERT_2_C Short Inter D-H..H-D       H2A      ..H68       .       2.12 Ang.
                                                  -x,1-y,-z  =      2_565 Check
PLAT906_ALERT_3_C Large K Value in the Analysis of Variance ......     21.211 Check
PLAT906_ALERT_3_C Large K Value in the Analysis of Variance ......      2.078 Check
PLAT906_ALERT_3_C Large K Value in the Analysis of Variance ......      4.530 Check
PLAT906_ALERT_3_C Large K Value in the Analysis of Variance ......      2.267 Check
PLAT911_ALERT_3_C Missing FCF Refl Between Thmin & STh/L=    0.600        484 Report
PLAT918_ALERT_3_C Reflection(s) with I(obs) much Smaller I(calc) .          1 Check
PLAT922_ALERT_1_C wR2 in the CIF and FCF Differ by ..............    -0.0038 Check
PLAT923_ALERT_1_C S    Values in the CIF and FCF Differ by .......     -0.011 Check
PLAT927_ALERT_1_C Reported and Calculated  wR2 Differ by .........    -0.0040 Check
PLAT939_ALERT_3_C Large Value of Not (SHELXL) Weight Optimized S .      25.96 Check
PLAT976_ALERT_2_C Check Calcd Resid. Dens.  1.02A   From N1           -0.52 eA-3
PLAT977_ALERT_2_C Check Negative Difference Density on H1A            -0.48 eA-3
PLAT977_ALERT_2_C Check Negative Difference Density on H3             -0.36 eA-3
PLAT977_ALERT_2_C Check Negative Difference Density on H20            -0.37 eA-3
PLAT977_ALERT_2_C Check Negative Difference Density on H43            -0.37 eA-3
PLAT977_ALERT_2_C Check Negative Difference Density on H58            -0.33 eA-3
PLAT977_ALERT_2_C Check Negative Difference Density on H59            -0.34 eA-3
PLAT977_ALERT_2_C Check Negative Difference Density on H61            -0.31 eA-3
PLAT977_ALERT_2_C Check Negative Difference Density on H68            -0.32 eA-3
```

Alert level G

```
PLAT002_ALERT_2_G Number of Distance or Angle Restraints on AtSite          4 Note
PLAT007_ALERT_5_G Number of Unrefined Donor-H Atoms ..............          6 Report
PLAT012_ALERT_1_G N.O.K.   _shelx_res_checksum Found in CIF ......     Please Check
PLAT066_ALERT_1_G Predicted and Reported Tmin&Tmax Range Identical          ? Check
PLAT172_ALERT_4_G The CIF-Embedded .res File Contains DFIX Records          2 Report
PLAT302_ALERT_4_G Anion/Solvent/Minor-Residue Disorder (Resd  3  )       100% Note
PLAT302_ALERT_4_G Anion/Solvent/Minor-Residue Disorder (Resd  4  )       100% Note
PLAT380_ALERT_4_G Incorrectly? Oriented X(sp2)-Methyl Moiety .....        C58 Check
PLAT722_ALERT_1_G Angle   Calc      114.00, Rep      112.60 Dev...       1.40 Degree
              N6      -C56     -H60        1.555  1.555  1.555     #  224 Check
PLAT790_ALERT_4_G Centre of Gravity not Within Unit Cell: Resd.  #          3 Note
              C5 H9 N O
PLAT790_ALERT_4_G Centre of Gravity not Within Unit Cell: Resd.  #          4 Note
              C5 H9 N O
PLAT790_ALERT_4_G Centre of Gravity not Within Unit Cell: Resd.  #          5 Note
              C H4 O
PLAT790_ALERT_4_G Centre of Gravity not Within Unit Cell: Resd.  #          6 Note
              C H4 O
PLAT860_ALERT_3_G Number of Least-Squares Restraints .............          2 Note
PLAT883_ALERT_1_G No Info/Value for _atom_sites_solution_primary .     Please Do !
PLAT912_ALERT_4_G Missing # of FCF Reflections Above STh/L=  0.600        251 Note
PLAT941_ALERT_3_G Average HKL Measurement Multiplicity ...........        1.8 Low
PLAT960_ALERT_3_G Number of Intensities with I  <   - 2*sig(I) ...          8 Check
PLAT978_ALERT_2_G Number C-C Bonds with Positive Residual Density.          0 Info
```

```
  1 ALERT level A = Most likely a serious problem - resolve or explain
  5 ALERT level B = A potentially serious problem, consider carefully
 33 ALERT level C = Check. Ensure it is not caused by an omission or oversight
 19 ALERT level G = General information/check it is not something unexpected

  7 ALERT type 1 CIF construction/syntax error, inconsistent or missing data
 18 ALERT type 2 Indicator that the structure model may be wrong or deficient
 22 ALERT type 3 Indicator that the structure quality may be low
 10 ALERT type 4 Improvement, methodology, query or suggestion
  1 ALERT type 5 Informative message, check
```

It is advisable to attempt to resolve as many as possible of the alerts in all categories. Often the minor alerts point to easily fixed oversights, errors and omissions in your CIF or refinement strategy, so attention to these fine details can be worthwhile. In order to resolve some of the more serious problems it may be necessary to carry out additional measurements or structure refinements. However, the purpose of your study may justify the reported deviations and the more serious of these should normally be commented upon in the discussion or experimental section of a paper or in the "special_details" fields of the CIF. checkCIF was carefully designed to identify outliers and unusual parameters, but every test has its limitations and alerts that are not important in a particular case may appear. Conversely, the absence of alerts does not guarantee there are no aspects of the results needing attention. It is up to the individual to critically assess their own results and, if necessary, seek expert advice.

Publication of your CIF in IUCr journals

A basic structural check has been run on your CIF. These basic checks will be run on all CIFs submitted for publication in IUCr journals (*Acta Crystallographica*, *Journal of Applied Crystallography*, *Journal of Synchrotron Radiation*); however, if you intend to submit to *Acta Crystallographica Section C* or *E* or *IUCrData*, you should make sure that full publication checks are run on the final version of your CIF prior to submission.

Publication of your CIF in other journals

Please refer to the *Notes for Authors* of the relevant journal for any special instructions relating to CIF submission.

Validation response form

Please find below a validation response form (VRF) that can be filled in and pasted into your CIF.

```
# start Validation Reply Form
_vrf_RINTA01_aPhCu3
;
PROBLEM: The value of Rint is greater than 0.12
RESPONSE: ...
;
_vrf_PLAT026_aPhCu3
;
PROBLEM: Ratio Observed / Unique Reflections (too) Low ..        39% Check
RESPONSE: ...
;
_vrf_PLAT084_aPhCu3
;
PROBLEM: High wR2 Value (i.e. > 0.25) ...................       0.37 Report
RESPONSE: ...
;
_vrf_PLAT417_aPhCu3
;
PROBLEM: Short Inter D-H..H-D       H1A      ..H68      .       2.06 Ang.
RESPONSE: ...
;
_vrf_PLAT910_aPhCu3
;
PROBLEM: Missing # of FCF Reflection(s) Below Theta(Min).         17 Note
RESPONSE: ...
;
_vrf_PLAT020_aPhCu3
```

```
;
PROBLEM: The Value of Rint is Greater Than 0.12 .........      0.157 Report
RESPONSE: ...
;
_vrf_PLAT082_aPhCu3
;
PROBLEM: High R1 Value ..................................       0.12 Report
RESPONSE: ...
;
_vrf_PLAT148_aPhCu3
;
PROBLEM: s.u. on the      a    - Axis is (Too) Large ....      0.020 Ang.
RESPONSE: ...
;
_vrf_PLAT149_aPhCu3
;
PROBLEM: s.u. on the   beta    Angle is Too Large .......       0.11 Degree
RESPONSE: ...
;
_vrf_PLAT202_aPhCu3
;
PROBLEM: Isotropic non-H Atoms in Anion/Solvent .........          1 Check
RESPONSE: ...
;
_vrf_PLAT234_aPhCu3
;
PROBLEM: Large Hirshfeld Difference C3       --C4       .       0.20 Ang.
RESPONSE: ...
;
_vrf_PLAT331_aPhCu3
;
PROBLEM: Small Aver Phenyl C-C Dist C18      --C23      .       1.37 Ang.
RESPONSE: ...
;
_vrf_PLAT341_aPhCu3
;
PROBLEM: Low Bond Precision on  C-C Bonds ..............    0.01412 Ang.
RESPONSE: ...
;
_vrf_PLAT906_aPhCu3
;
PROBLEM: Large K Value in the Analysis of Variance ......     21.211 Check
RESPONSE: ...
;
_vrf_PLAT911_aPhCu3
;
PROBLEM: Missing FCF Refl Between Thmin & STh/L=    0.600        484 Report
RESPONSE: ...
;
_vrf_PLAT918_aPhCu3
;
PROBLEM: Reflection(s) with I(obs) much Smaller I(calc) .          1 Check
RESPONSE: ...
;
_vrf_PLAT922_aPhCu3
;
PROBLEM: wR2 in the CIF and FCF Differ by ..............    -0.0038 Check
RESPONSE: ...
;
_vrf_PLAT923_aPhCu3
;
PROBLEM: S    Values in the CIF and FCF Differ by .......     -0.011 Check
```

```
RESPONSE: ...
;
_vrf_PLAT927_aPhCu3
;
PROBLEM: Reported and Calculated  wR2 Differ by .........    -0.0040 Check
RESPONSE: ...
;
_vrf_PLAT939_aPhCu3
;
PROBLEM: Large Value of Not (SHELXL) Weight Optimized S .      25.96 Check
RESPONSE: ...
;
_vrf_PLAT976_aPhCu3
;
PROBLEM: Check Calcd Resid. Dens.  1.02A   From N1             -0.52 eA-3
RESPONSE: ...
;
_vrf_PLAT977_aPhCu3
;
PROBLEM: Check Negative Difference Density on H1A              -0.48 eA-3
RESPONSE: ...
;
# end Validation Reply Form
```

PLATON version of 05/12/2020; check.def file version of 05/12/2020

Datablock aPhCu3 - ellipsoid plot

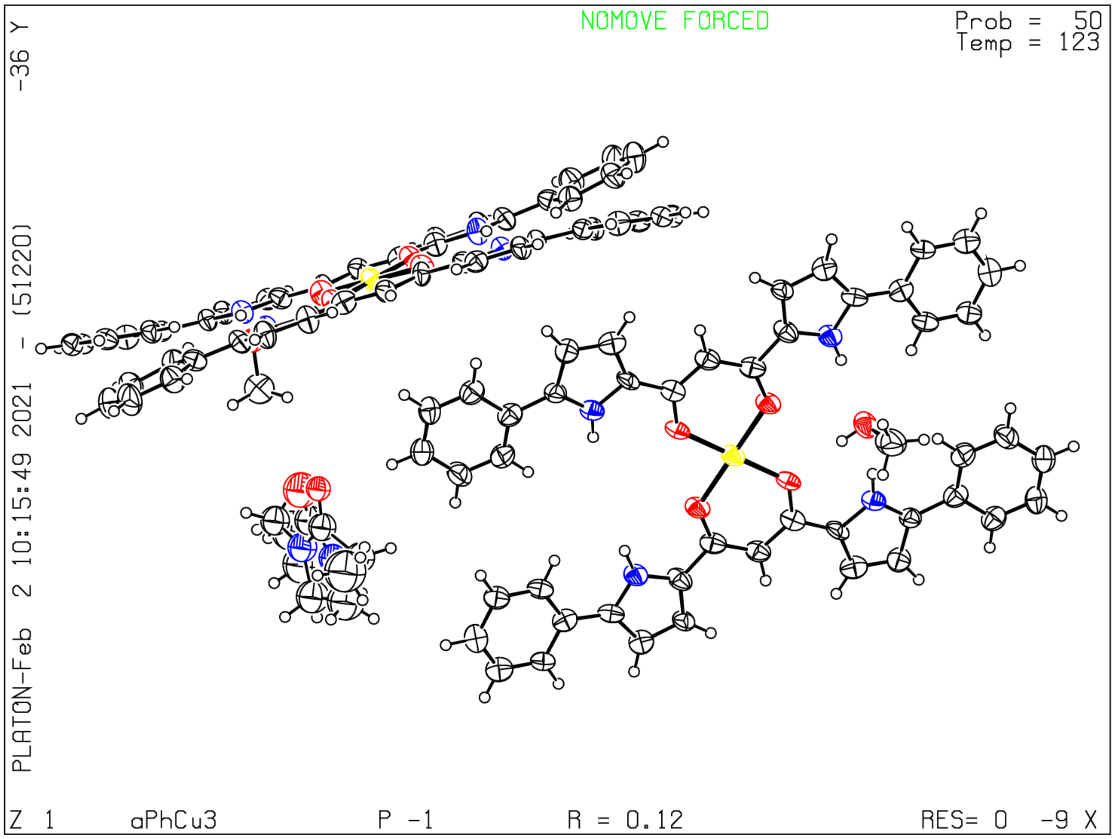

checkCIF/PLATON report

Structure factors have been supplied for datablock(s) 90425-2_yh

THIS REPORT IS FOR GUIDANCE ONLY. IF USED AS PART OF A REVIEW PROCEDURE FOR PUBLICATION, IT SHOULD NOT REPLACE THE EXPERTISE OF AN EXPERIENCED CRYSTALLOGRAPHIC REFEREE.

No syntax errors found. CIF dictionary Interpreting this report

Datablock: 90425-2_yh

Bond precision:	C-C = 0.0081 A		Wavelength=0.71075
Cell:	a=10.962(6)	b=9.650(6)	c=20.748(14)
	alpha=90	beta=93.83(2)	gamma=90
Temperature:	123 K		

	Calculated	Reported
Volume	2190(2)	2190(2)
Space group	P 2/c	P 2/c
Hall group	-P 2yc	-P 2yc
Moiety formula	C50 H42 Cu N4 O8, 2(C H4 O)	C50 H42 Cu N4 O8, 2(C H4 O)
Sum formula	C52 H50 Cu N4 O10	C52 H50 Cu N4 O10
Mr	954.51	954.50
Dx,g cm-3	1.447	1.448
Z	2	2
Mu (mm-1)	0.567	0.567
F000	998.0	998.0
F000’	999.08	
h,k,lmax	14,12,26	14,12,26
Nref	5026	4974
Tmin,Tmax	0.903,0.945	0.895,0.945
Tmin’	0.893	

Correction method= # Reported T Limits: Tmin=0.895 Tmax=0.945
AbsCorr = NONE

Data completeness= 0.990 Theta(max)= 27.480

R(reflections)= 0.0951(2389) wR2(reflections)= 0.2499(4974)

S = 1.035 Npar= 308

```
The following ALERTS were generated. Each ALERT has the format
       test-name_ALERT_alert-type_alert-level.
Click on the hyperlinks for more details of the test.
```

Alert level B

```
RINTA01_ALERT_3_B  The value of Rint is greater than 0.18
            Rint given   0.183
PLAT020_ALERT_3_B The Value of Rint is Greater Than 0.12 .........      0.183 Report
PLAT417_ALERT_2_B Short Inter D-H..H-D       H14      ..H25      .       2.09 Ang.
                                                       x,y,z  =      1_555 Check
```

Alert level C

```
PLAT026_ALERT_3_C Ratio Observed / Unique Reflections (too) Low ..        48% Check
PLAT341_ALERT_3_C Low Bond Precision on  C-C Bonds ...............    0.00813 Ang.
PLAT906_ALERT_3_C Large K Value in the Analysis of Variance ......     33.092 Check
PLAT906_ALERT_3_C Large K Value in the Analysis of Variance ......      2.360 Check
PLAT906_ALERT_3_C Large K Value in the Analysis of Variance ......      6.571 Check
PLAT906_ALERT_3_C Large K Value in the Analysis of Variance ......      2.798 Check
PLAT906_ALERT_3_C Large K Value in the Analysis of Variance ......      2.096 Check
PLAT910_ALERT_3_C Missing # of FCF Reflection(s) Below Theta(Min).          9 Note
PLAT934_ALERT_3_C Number of (Iobs-Icalc)/Sigma(W) > 10 Outliers ..          1 Check
```

Alert level G

```
PLAT007_ALERT_5_G Number of Unrefined Donor-H Atoms ..............          3 Report
PLAT066_ALERT_1_G Predicted and Reported Tmin&Tmax Range Identical          ? Check
PLAT790_ALERT_4_G Centre of Gravity not Within Unit Cell: Resd.  #          2 Note
             C H4 O
PLAT883_ALERT_1_G No Info/Value for _atom_sites_solution_primary .     Please Do !
PLAT912_ALERT_4_G Missing # of FCF Reflections Above STh/L=  0.600         42 Note
PLAT941_ALERT_3_G Average HKL Measurement Multiplicity ...........        3.8 Low
PLAT978_ALERT_2_G Number C-C Bonds with Positive Residual Density.          2 Info
```

```
   0 ALERT level A = Most likely a serious problem - resolve or explain
   3 ALERT level B = A potentially serious problem, consider carefully
   9 ALERT level C = Check. Ensure it is not caused by an omission or oversight
   7 ALERT level G = General information/check it is not something unexpected

   2 ALERT type 1 CIF construction/syntax error, inconsistent or missing data
   2 ALERT type 2 Indicator that the structure model may be wrong or deficient
  12 ALERT type 3 Indicator that the structure quality may be low
   2 ALERT type 4 Improvement, methodology, query or suggestion
   1 ALERT type 5 Informative message, check
```

Validation response form

Please find below a validation response form (VRF) that can be filled in and pasted into your CIF.

```
# start Validation Reply Form
_vrf_RINTA01_90425-2_yh
;
PROBLEM: The value of Rint is greater than 0.18
RESPONSE: ...
;
_vrf_PLAT020_90425-2_yh
;
PROBLEM: The Value of Rint is Greater Than 0.12 .........      0.183 Report
```

```
RESPONSE: ...
;
_vrf_PLAT417_90425-2_yh
;
PROBLEM: Short Inter D-H..H-D       H14      ..H25      .       2.09 Ang.
RESPONSE: ...
;
_vrf_PLAT026_90425-2_yh
;
PROBLEM: Ratio Observed / Unique Reflections (too) Low ..        48% Check
RESPONSE: ...
;
_vrf_PLAT341_90425-2_yh
;
PROBLEM: Low Bond Precision on  C-C Bonds ...............    0.00813 Ang.
RESPONSE: ...
;
_vrf_PLAT906_90425-2_yh
;
PROBLEM: Large K Value in the Analysis of Variance ......     33.092 Check
RESPONSE: ...
;
_vrf_PLAT910_90425-2_yh
;
PROBLEM: Missing # of FCF Reflection(s) Below Theta(Min).          9 Note
RESPONSE: ...
;
_vrf_PLAT934_90425-2_yh
;
PROBLEM: Number of (Iobs-Icalc)/Sigma(W) > 10 Outliers ..          1 Check
RESPONSE: ...
;
# end Validation Reply Form
```

It is advisable to attempt to resolve as many as possible of the alerts in all categories. Often the minor alerts point to easily fixed oversights, errors and omissions in your CIF or refinement strategy, so attention to these fine details can be worthwhile. In order to resolve some of the more serious problems it may be necessary to carry out additional measurements or structure refinements. However, the purpose of your study may justify the reported deviations and the more serious of these should normally be commented upon in the discussion or experimental section of a paper or in the "special_details" fields of the CIF. checkCIF was carefully designed to identify outliers and unusual parameters, but every test has its limitations and alerts that are not important in a particular case may appear. Conversely, the absence of alerts does not guarantee there are no aspects of the results needing attention. It is up to the individual to critically assess their own results and, if necessary, seek expert advice.

Publication of your CIF in IUCr journals

A basic structural check has been run on your CIF. These basic checks will be run on all CIFs submitted for publication in IUCr journals (*Acta Crystallographica*, *Journal of Applied Crystallography*, *Journal of Synchrotron Radiation*); however, if you intend to submit to *Acta Crystallographica Section C* or *E* or *IUCrData*, you should make sure that full publication checks are run on the final version of your CIF prior to submission.

Publication of your CIF in other journals

Please refer to the *Notes for Authors* of the relevant journal for any special instructions relating to CIF submission.

PLATON version of 05/12/2020; check.def file version of 05/12/2020

Datablock 90425-2_yh - ellipsoid plot

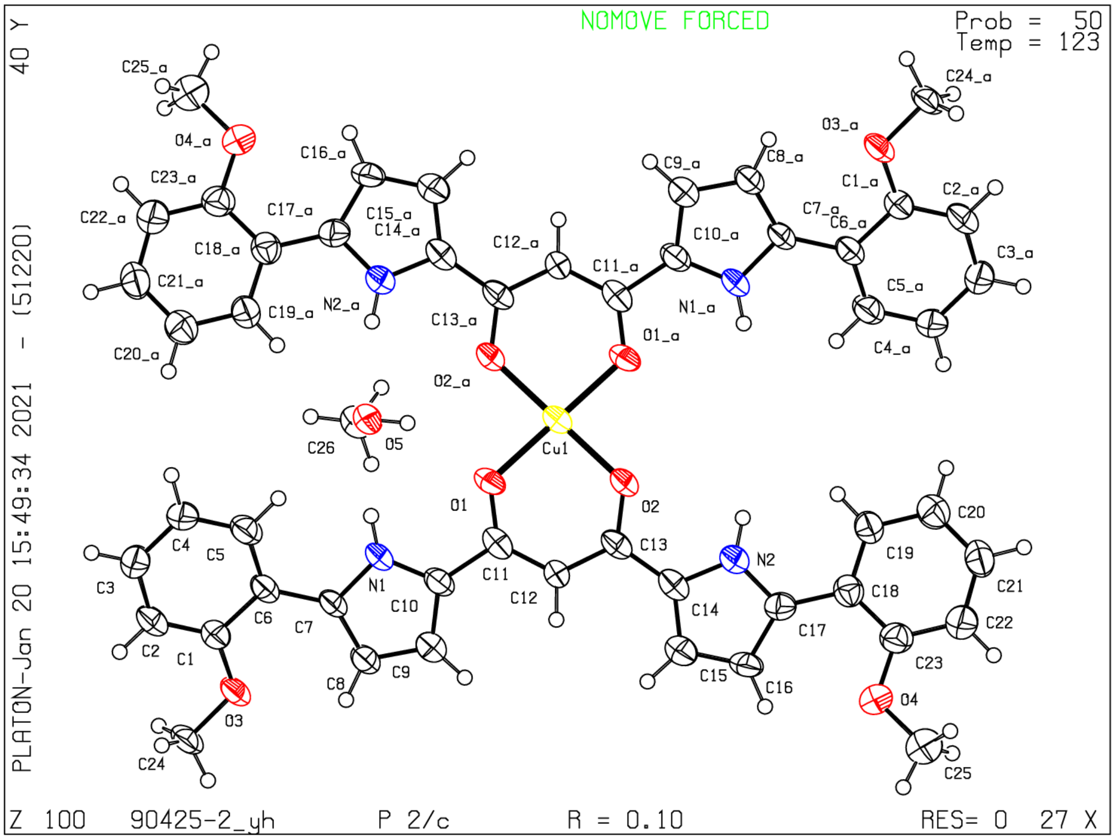

checkCIF/PLATON report

Structure factors have been supplied for datablock(s) 90415-2_sqd

THIS REPORT IS FOR GUIDANCE ONLY. IF USED AS PART OF A REVIEW PROCEDURE FOR PUBLICATION, IT SHOULD NOT REPLACE THE EXPERTISE OF AN EXPERIENCED CRYSTALLOGRAPHIC REFEREE.

No syntax errors found. CIF dictionary Interpreting this report

Datablock: 90415-2_sqd

```
Bond precision:  C-C = 0.0040 A          Wavelength=0.71075

Cell:            a=9.714(3)     b=15.320(6)          c=32.192(9)
                 alpha=90       beta=91.107(11)      gamma=90
Temperature:     123 K
```

	Calculated	Reported
Volume	4790(3)	4790(3)
Space group	P 21/c	P 21/c
Hall group	-P 2ybc	-P 2ybc
Moiety formula	C50 H42 Cu N4 O8, 2(C H4 O) [+ solvent]	C50 H42 Cu N4 O8, 2(C H4 O), C5 H9 N O
Sum formula	C52 H50 Cu N4 O10 [+ solvent]	C57 H59 Cu N5 O11
Mr	954.51	1053.63
Dx,g cm-3	1.324	1.461
Z	4	4
Mu (mm-1)	0.519	0.528
F000	1996.0	2212.0
F000’	1998.15	
h,k,lmax	12,19,41	12,19,41
Nref	10988	10902
Tmin,Tmax	0.776,0.900	0.895,0.945
Tmin’	0.728	

```
Correction method= # Reported T Limits: Tmin=0.895 Tmax=0.945
AbsCorr = NONE

Data completeness= 0.992          Theta(max)= 27.483

R(reflections)= 0.0553( 6620)     wR2(reflections)= 0.1490( 10902)

S = 0.984                  Npar= 612
```

The following ALERTS were generated. Each ALERT has the format
test-name_ALERT_alert-type_alert-level.
Click on the hyperlinks for more details of the test.

Alert level B

```
PLAT417_ALERT_2_B Short Inter D-H..H-D       H4A      ..H10A     .       2.08 Ang.
                                                     x,y,z  =      1_555 Check
PLAT910_ALERT_3_B Missing # of FCF Reflection(s) Below Theta(Min).         17 Note
PLAT990_ALERT_1_B Deprecated .res/.hkl Input Style SQUEEZE Job ...          ! Note
```

Alert level C

```
PLAT057_ALERT_3_C Correction for Absorption Required   RT(exp) ...       1.16 Do !
PLAT417_ALERT_2_C Short Inter D-H..H-D       H2       ..H9A      .       2.12 Ang.
                                                     x,y,z  =      1_555 Check
PLAT906_ALERT_3_C Large K Value in the Analysis of Variance ......      5.854 Check
PLAT911_ALERT_3_C Missing FCF Refl Between Thmin & STh/L=    0.600         36 Report
```

Alert level G

```
FORMU01_ALERT_2_G  There is a discrepancy between the atom counts in the
            _chemical_formula_sum and the formula from the _atom_site* data.
            Atom count from _chemical_formula_sum:C57 H59 Cu1 N5 O11
            Atom count from the _atom_site data:  C52 H50 Cu1 N4 O10
CELLZ01_ALERT_1_G Difference between formula and atom_site contents detected.
CELLZ01_ALERT_1_G ALERT: Large difference may be due to a
            symmetry error - see SYMMG tests
           From the CIF: _cell_formula_units_Z    4
           From the CIF: _chemical_formula_sum  C57 H59 Cu N5 O11
           TEST: Compare cell contents of formula and atom_site data

           atom    Z*formula  cif sites diff
           C        228.00    208.00   20.00
           H        236.00    200.00   36.00
           Cu         4.00      4.00    0.00
           N         20.00     16.00    4.00
           O         44.00     40.00    4.00
PLAT007_ALERT_5_G Number of Unrefined Donor-H Atoms ..............          6 Report
PLAT041_ALERT_1_G Calc. and Reported SumFormula    Strings  Differ     Please Check
PLAT068_ALERT_1_G Reported F000 Differs from Calcd (or Missing)...     Please Check
PLAT605_ALERT_4_G Largest Solvent Accessible VOID in the Structure        218 A**3
PLAT869_ALERT_4_G ALERTS Related to the Use of SQUEEZE Suppressed           ! Info
PLAT883_ALERT_1_G No Info/Value for _atom_sites_solution_primary .     Please Do !
PLAT912_ALERT_4_G Missing # of FCF Reflections Above STh/L=  0.600         34 Note
PLAT941_ALERT_3_G Average HKL Measurement Multiplicity ...........        3.8 Low
PLAT961_ALERT_5_G Dataset Contains no Negative Intensities .......     Please Check
PLAT978_ALERT_2_G Number C-C Bonds with Positive Residual Density.          3 Info
```

---

0 **ALERT level A** = Most likely a serious problem - resolve or explain
3 **ALERT level B** = A potentially serious problem, consider carefully
4 **ALERT level C** = Check. Ensure it is not caused by an omission or oversight
13 **ALERT level G** = General information/check it is not something unexpected

6 ALERT type 1 CIF construction/syntax error, inconsistent or missing data
4 ALERT type 2 Indicator that the structure model may be wrong or deficient
5 ALERT type 3 Indicator that the structure quality may be low
3 ALERT type 4 Improvement, methodology, query or suggestion
2 ALERT type 5 Informative message, check

Validation response form

Please find below a validation response form (VRF) that can be filled in and pasted into your CIF.

```
# start Validation Reply Form
_vrf_PLAT417_90415-2_sqd
;
PROBLEM: Short Inter D-H..H-D       H4A      ..H10A     .       2.08 Ang.
RESPONSE: ...
;
_vrf_PLAT910_90415-2_sqd
;
PROBLEM: Missing # of FCF Reflection(s) Below Theta(Min).         17 Note
RESPONSE: ...
;
_vrf_PLAT990_90415-2_sqd
;
PROBLEM: Deprecated .res/.hkl Input Style SQUEEZE Job ...          ! Note
RESPONSE: ...
;
_vrf_PLAT057_90415-2_sqd
;
PROBLEM: Correction for Absorption Required   RT(exp) ...       1.16 Do !
RESPONSE: ...
;
_vrf_PLAT906_90415-2_sqd
;
PROBLEM: Large K Value in the Analysis of Variance ......      5.854 Check
RESPONSE: ...
;
_vrf_PLAT911_90415-2_sqd
;
PROBLEM: Missing FCF Refl Between Thmin & STh/L=    0.600         36 Report
RESPONSE: ...
;
# end Validation Reply Form
```

It is advisable to attempt to resolve as many as possible of the alerts in all categories. Often the minor alerts point to easily fixed oversights, errors and omissions in your CIF or refinement strategy, so attention to these fine details can be worthwhile. In order to resolve some of the more serious problems it may be necessary to carry out additional measurements or structure refinements. However, the purpose of your study may justify the reported deviations and the more serious of these should normally be commented upon in the discussion or experimental section of a paper or in the "special_details" fields of the CIF. checkCIF was carefully designed to identify outliers and unusual parameters, but every test has its limitations and alerts that are not important in a particular case may appear. Conversely, the absence of alerts does not guarantee there are no aspects of the results needing attention. It is up to the individual to critically assess their own results and, if necessary, seek expert advice.

Publication of your CIF in IUCr journals

A basic structural check has been run on your CIF. These basic checks will be run on all CIFs submitted for publication in IUCr journals (*Acta Crystallographica*, *Journal of Applied Crystallography*, *Journal of Synchrotron Radiation*); however, if you intend to submit to *Acta Crystallographica Section C* or *E* or *IUCrData*, you should make sure that full publication checks are run on the final version of your CIF prior to submission.

Publication of your CIF in other journals

Please refer to the *Notes for Authors* of the relevant journal for any special instructions relating to CIF submission.

PLATON version of 05/12/2020; check.def file version of 05/12/2020

Datablock 90415-2_sqd - ellipsoid plot

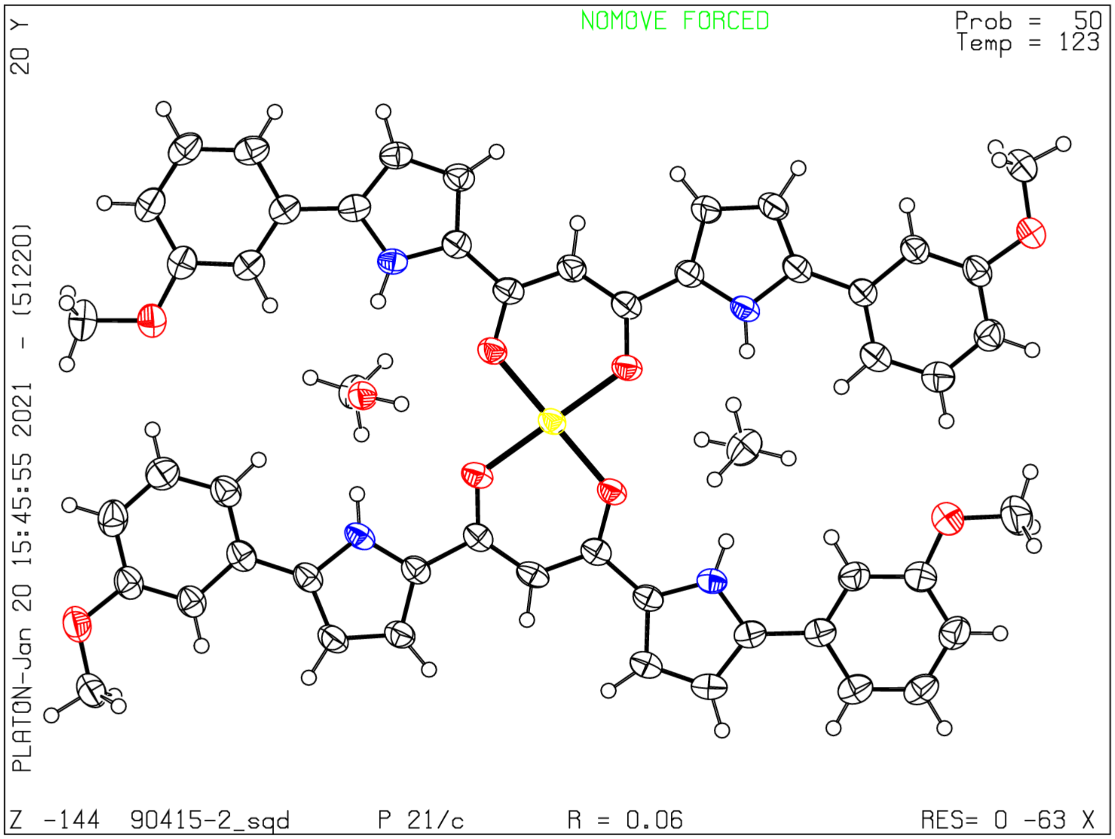

checkCIF/PLATON report

Structure factors have been supplied for datablock(s) 71203mae_a_sqd2

THIS REPORT IS FOR GUIDANCE ONLY. IF USED AS PART OF A REVIEW PROCEDURE FOR PUBLICATION, IT SHOULD NOT REPLACE THE EXPERTISE OF AN EXPERIENCED CRYSTALLOGRAPHIC REFEREE.

No syntax errors found. CIF dictionary Interpreting this report

Datablock: 71203mae_a_sqd2

Bond precision: C-C = 0.0030 A Wavelength=0.71075

Cell:	a=12.778(3)	b=21.393(5)	c=11.915(4)
	alpha=90	beta=108.825(11)	gamma=90
Temperature:	123 K		

	Calculated	Reported
Volume	3082.9(15)	3082.9(14)
Space group	P 21/c	P 21/c
Hall group	-P 2ybc	-P 2ybc
Moiety formula	C58 H58 Cu N4 O16, 2(C H4 O) [+ solvent]	C58 H58 Cu N4 O16, 2(C H4 O), C H2 Cl2
Sum formula	C60 H66 Cu N4 O18 [+ solvent]	C61 H68 Cl2 Cu N4 O18
Mr	1194.72	1279.63
Dx,g cm-3	1.287	1.378
Z	2	2
Mu (mm-1)	0.427	0.515
F000	1254.0	1338.0
F000’	1255.27	
h,k,lmax	16,27,15	16,27,15
Nref	7079	7069
Tmin,Tmax	0.884,0.950	0.820,0.950
Tmin’	0.814	

Correction method= # Reported T Limits: Tmin=0.820 Tmax=0.950
AbsCorr = NONE

Data completeness= 0.999 Theta(max)= 27.484

R(reflections)= 0.0446(5121) wR2(reflections)= 0.1143(7069)

S = 1.055 Npar= 384

```
The following ALERTS were generated. Each ALERT has the format
       test-name_ALERT_alert-type_alert-level.
Click on the hyperlinks for more details of the test.
```

Alert level B

```
PLAT417_ALERT_2_B Short Inter D-H..H-D       H32      ..H33      .       2.02 Ang.
                                                   x,y,z  =      1_555 Check
PLAT990_ALERT_1_B Deprecated .res/.hkl Input Style SQUEEZE Job ...          ! Note
```

Alert level C

```
PLAT906_ALERT_3_C Large K Value in the Analysis of Variance ......      5.537 Check
PLAT910_ALERT_3_C Missing # of FCF Reflection(s) Below Theta(Min).          8 Note
```

Alert level G

```
FORMU01_ALERT_2_G  There is a discrepancy between the atom counts in the
            _chemical_formula_sum and the formula from the _atom_site* data.
            Atom count from _chemical_formula_sum:C61 H68 Cl2 Cu1 N4 O18
            Atom count from the _atom_site data:  C60 H66 Cu1 N4 O18
CELLZ01_ALERT_1_G Difference between formula and atom_site contents detected.
CELLZ01_ALERT_1_G ALERT: Large difference may be due to a
            symmetry error - see SYMMG tests
           From the CIF: _cell_formula_units_Z    2
           From the CIF: _chemical_formula_sum  C61 H68 Cl2 Cu N4 O18
           TEST: Compare cell contents of formula and atom_site data

           atom    Z*formula  cif sites diff
           C        122.00    120.00    2.00
           H        136.00    132.00    4.00
           Cl         4.00      0.00    4.00
           Cu         2.00      2.00    0.00
           N          8.00      8.00    0.00
           O         36.00     36.00    0.00
PLAT004_ALERT_5_G Polymeric Structure Found with Maximum Dimension          2 Info
PLAT007_ALERT_5_G Number of Unrefined Donor-H Atoms .............          3 Report
PLAT041_ALERT_1_G Calc. and Reported SumFormula    Strings  Differ     Please Check
PLAT051_ALERT_1_G Mu(calc) and Mu(CIF) Ratio Differs from 1.0 by .      17.18 %
PLAT066_ALERT_1_G Predicted and Reported Tmin&Tmax Range Identical          ? Check
PLAT068_ALERT_1_G Reported F000 Differs from Calcd (or Missing)...     Please Check
PLAT605_ALERT_4_G Largest Solvent Accessible VOID in the Structure        170 A**3
PLAT794_ALERT_5_G Tentative Bond Valency for Cu1       (II)      .       2.22 Info
PLAT869_ALERT_4_G ALERTS Related to the Use of SQUEEZE Suppressed           ! Info
PLAT883_ALERT_1_G No Info/Value for _atom_sites_solution_primary .     Please Do !
PLAT912_ALERT_4_G Missing # of FCF Reflections Above STh/L=  0.600          2 Note
PLAT941_ALERT_3_G Average HKL Measurement Multiplicity ...........        4.2 Low
PLAT961_ALERT_5_G Dataset Contains no Negative Intensities .......     Please Check
PLAT978_ALERT_2_G Number C-C Bonds with Positive Residual Density.          8 Info
```

```
   0 ALERT level A = Most likely a serious problem - resolve or explain
   2 ALERT level B = A potentially serious problem, consider carefully
   2 ALERT level C = Check. Ensure it is not caused by an omission or oversight
  17 ALERT level G = General information/check it is not something unexpected

   8 ALERT type 1 CIF construction/syntax error, inconsistent or missing data
   3 ALERT type 2 Indicator that the structure model may be wrong or deficient
   3 ALERT type 3 Indicator that the structure quality may be low
   3 ALERT type 4 Improvement, methodology, query or suggestion
   4 ALERT type 5 Informative message, check
```

Validation response form

Please find below a validation response form (VRF) that can be filled in and pasted into your CIF.

```
# start Validation Reply Form
_vrf_PLAT417_71203mae_a_sqd2
;
PROBLEM: Short Inter D-H..H-D       H32      ..H33      .       2.02 Ang.
RESPONSE: ...
;
_vrf_PLAT990_71203mae_a_sqd2
;
PROBLEM: Deprecated .res/.hkl Input Style SQUEEZE Job ...          ! Note
RESPONSE: ...
;
_vrf_PLAT906_71203mae_a_sqd2
;
PROBLEM: Large K Value in the Analysis of Variance ......      5.537 Check
RESPONSE: ...
;
_vrf_PLAT910_71203mae_a_sqd2
;
PROBLEM: Missing # of FCF Reflection(s) Below Theta(Min).          8 Note
RESPONSE: ...
;
# end Validation Reply Form
```

It is advisable to attempt to resolve as many as possible of the alerts in all categories. Often the minor alerts point to easily fixed oversights, errors and omissions in your CIF or refinement strategy, so attention to these fine details can be worthwhile. In order to resolve some of the more serious problems it may be necessary to carry out additional measurements or structure refinements. However, the purpose of your study may justify the reported deviations and the more serious of these should normally be commented upon in the discussion or experimental section of a paper or in the "special_details" fields of the CIF. checkCIF was carefully designed to identify outliers and unusual parameters, but every test has its limitations and alerts that are not important in a particular case may appear. Conversely, the absence of alerts does not guarantee there are no aspects of the results needing attention. It is up to the individual to critically assess their own results and, if necessary, seek expert advice.

Publication of your CIF in IUCr journals

A basic structural check has been run on your CIF. These basic checks will be run on all CIFs submitted for publication in IUCr journals (*Acta Crystallographica*, *Journal of Applied Crystallography*, *Journal of Synchrotron Radiation*); however, if you intend to submit to *Acta Crystallographica Section C* or *E* or *IUCrData*, you should make sure that full publication checks are run on the final version of your CIF prior to submission.

Publication of your CIF in other journals

Please refer to the *Notes for Authors* of the relevant journal for any special instructions relating to CIF submission.

PLATON version of 05/12/2020; check.def file version of 05/12/2020

Datablock 71203mae_a_sqd2 - ellipsoid plot

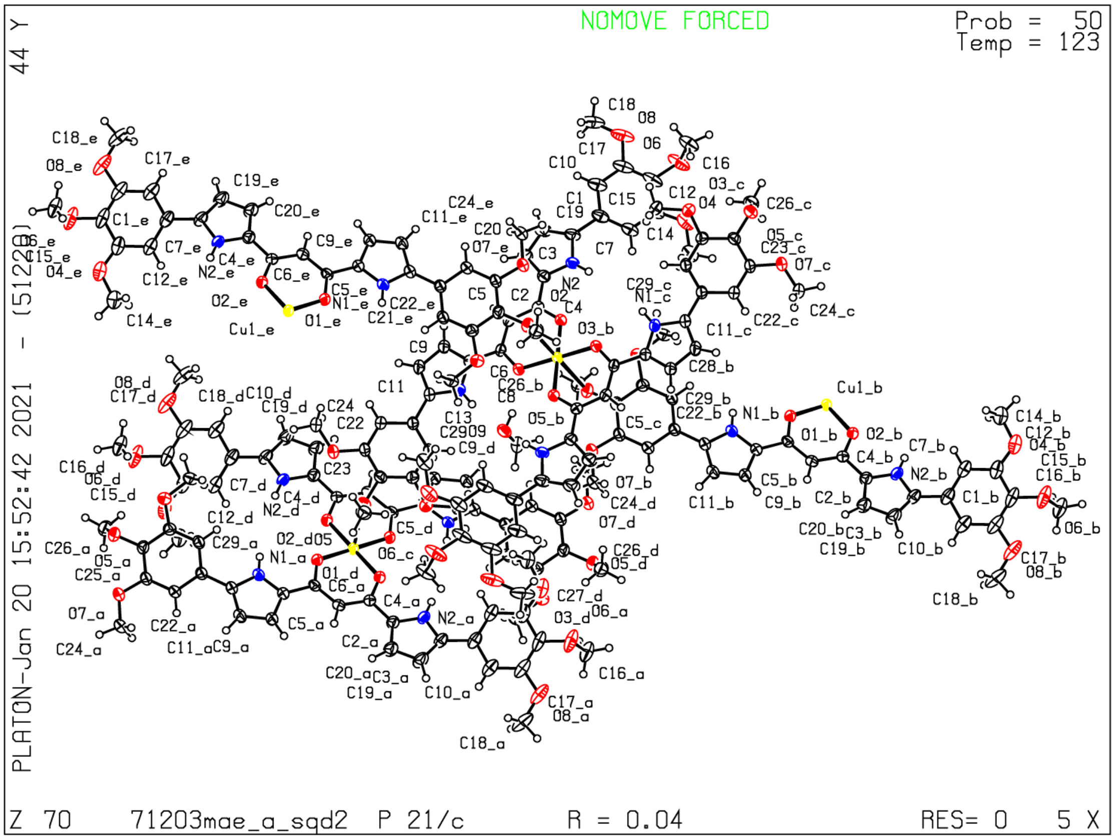

Supplement: Supplementary file 1 [file molecules-26-00861-s001.zip › Cu-diketone_HMaeda_SI/Cu-diketone_HMaeda_checkcif.pdf]
