# Supplementary material for: Supramolecular Assemblies of Dipyrrolyldiketone CuII Complexes
Source: Molecules. 2021 Feb 6;26(4):861. doi: 10.3390/molecules26040861 (PMC7914842; doi:10.3390/molecules26040861)
Supplement: Supplementary file 1 [file molecules-26-00861-s001.zip › Cu-diketone_HMaeda_SI/Cu-diketone_HMaeda_SI.pdf]

Supplementary Materials

Supramolecular Assemblies of Dipyrrolyldiketone Cu^{II} Complexes

Yohei Haketa and Hiromitsu Maeda*

Department of Applied Chemistry, College of Life Sciences, Ritsumeikan University, Kusatsu 525–8577, Japan, Fax: +81 77 561 2659; Tel: +81 77 561 5969; E-mail: maedahir@ph.ritsumei.ac.jp

Table of contents

1. Synthetic scheme and spectroscopic data S2
Figure S1 Synthesis of dipyrrolyldiketone Cu^{II} complexes. S2
Figure S2–5 ^{1}H NMR spectra. S2
Figure S6 UV/vis absorption spectrum. S4
2. X-ray crystallographic data S5
Figure S7–12 Ortep drawings of single-crystal X-ray structures. S6
Figure S13–18 Packing diagrams of single-crystal X-ray structures. S9
Figure S19 Hirshfeld surface. S15
3. Theoretical studies S16
Figure S20 Optimized structures. S16
Figure S21,22 Molecular orbitals and TD-DFT calculations. S17
Cartesian coordination of optimized structures S18
3. Assembled behaviors S20
Figure S23 DSC thermograms. S20
Figure S24 POM image. S20
Figure S25–28 XRD and packing diagrams. S21

1. Synthetic scheme and spectroscopic data

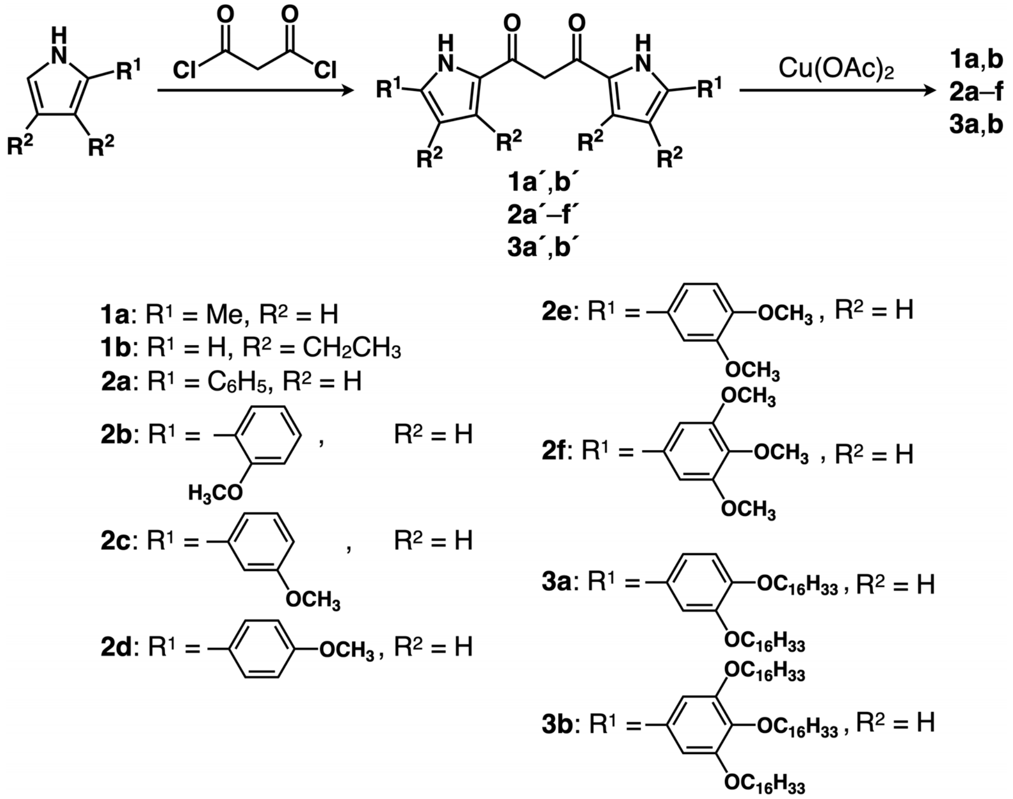

Figure S1 Synthesis of dipyrrolyldiketone Cu^{II} complexes.

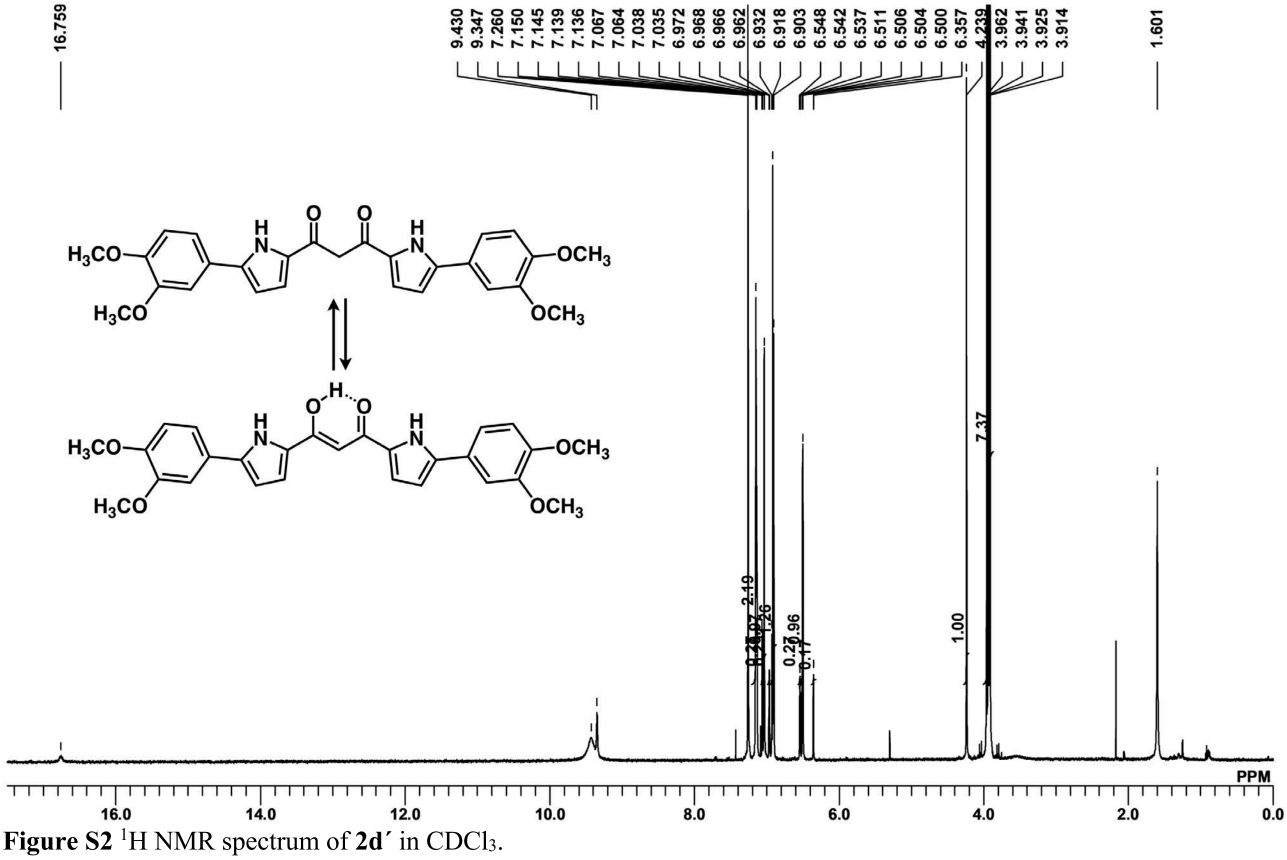

Figure S2 ^{1}H NMR spectrum of **2d´** in $CDCl_3$.

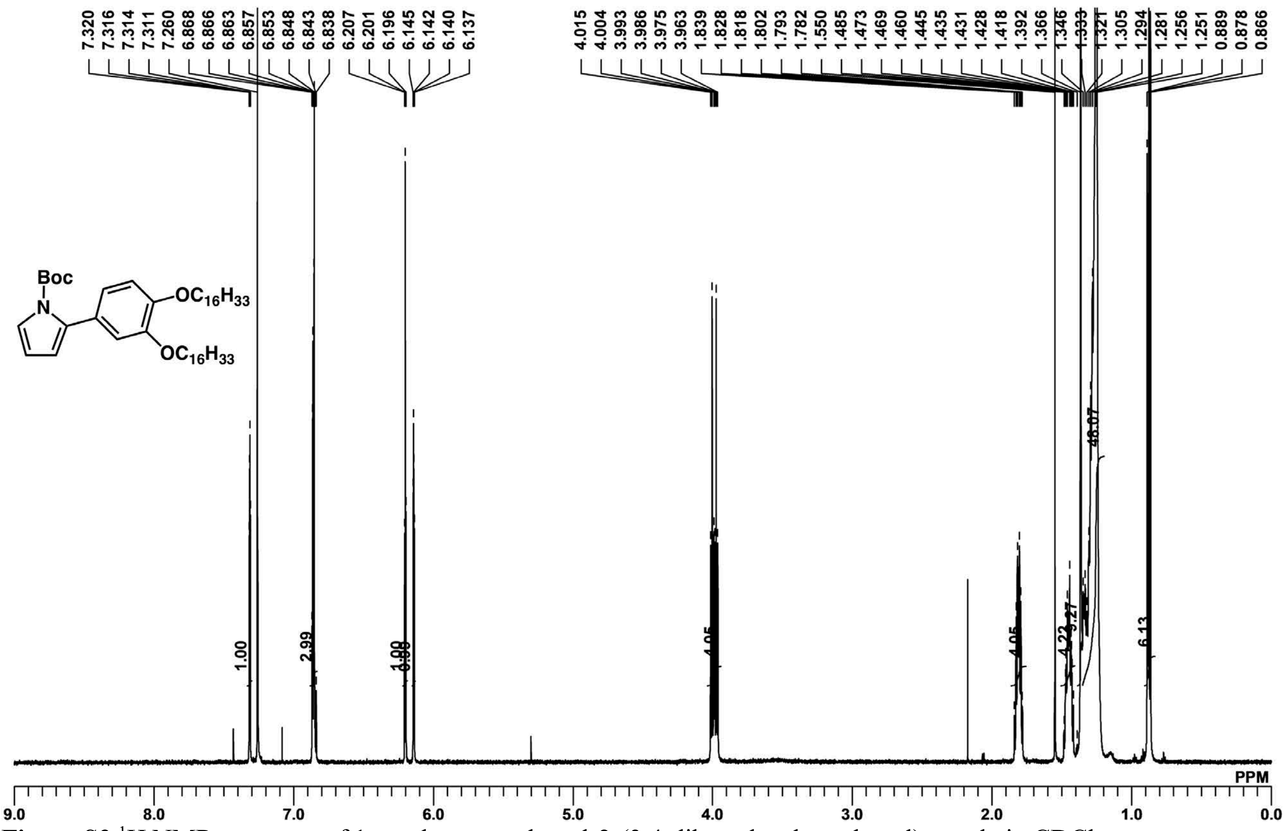

Figure S3 ^{1}H NMR spectrum of 1-*tert*-butoxycarbonyl-2-(3,4-dihexadecyloxyphenyl)pyrrole in $CDCl_3$.

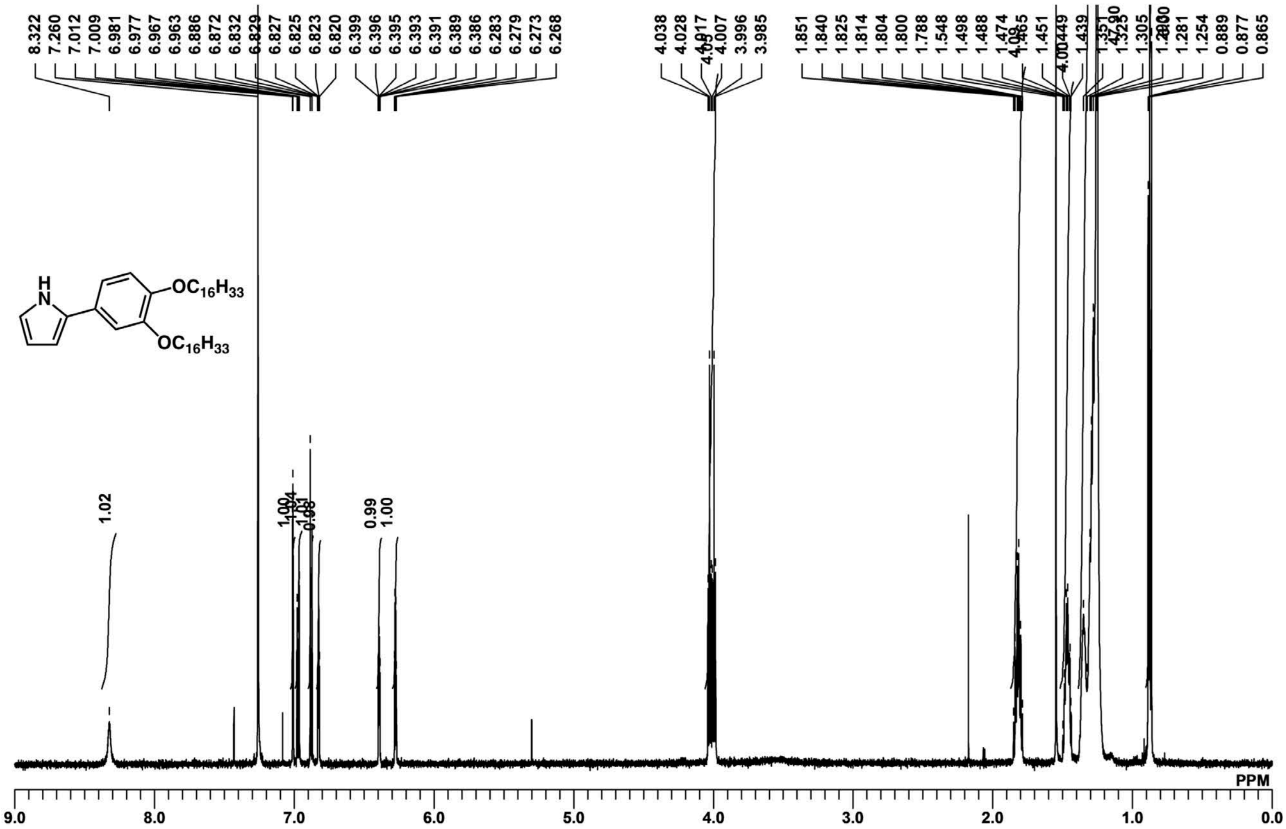

Figure S4 ^{1}H NMR spectrum of 2-(3,4-dihexadecyloxyphenyl)pyrrole in $CDCl_3$.

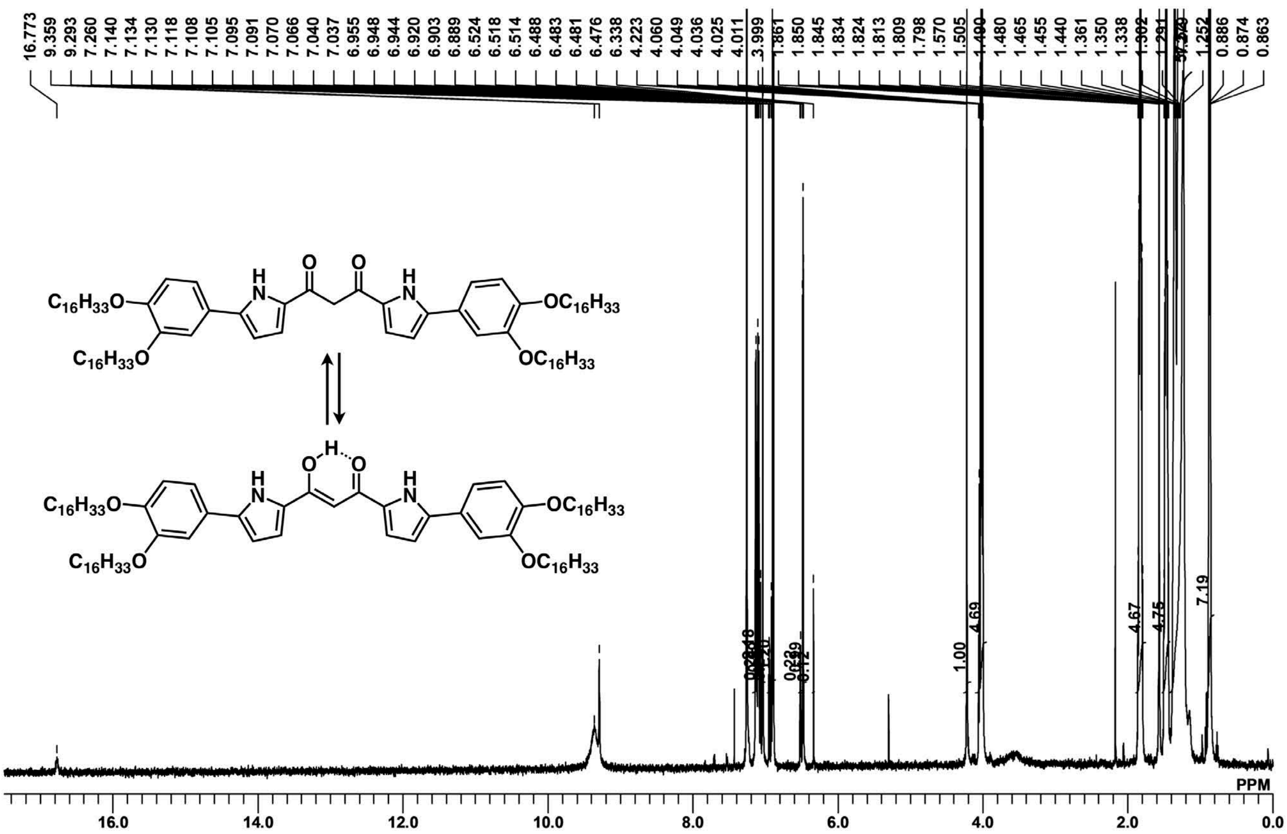

Figure S5 ^{1}H NMR spectrum of **3a′** in $CDCl_3$.

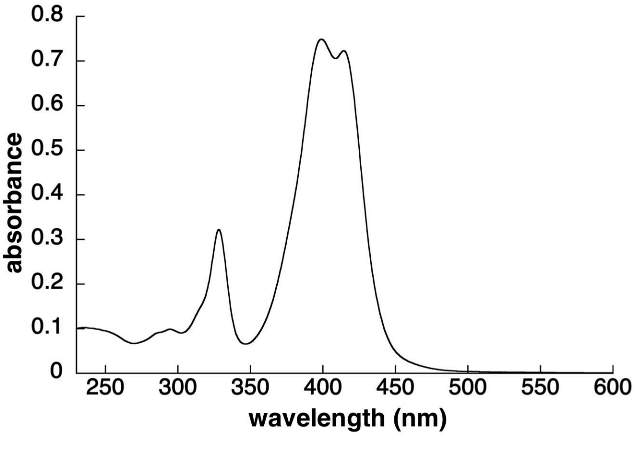

Figure S6 UV/vis absorption spectrum of **1a** in CH_2Cl_2 (1×10^{-5} M).

2. X-ray crystallographic data

Table S1 Crystallographic details.

	**1a**	**1b**	**2a**	**2b**	**2c**
formula	$C_{26}H_{26}CuN_4O_4\cdot 2H_2O$	$C_{38}H_{50}N_4O_4Cu\cdot 2CHCl_3$	$C_{46}H_{34}N_4O_4Cu\cdot C_5H_9NO\cdot 2CH_4O$	$C_{50}H_{42}N_4O_8Cu\cdot 2CH_4O$	$C_{50}H_{42}N_4O_8Cu\cdot 2CH_4O\cdot C_5H_9NO$
fw	573.13	929.10	933.52	954.50	1053.63
crystal size, mm	0.30 × 0.10 × 0.05	0.50 × 0.30 × 0.20	0.10 × 0.10 × 0.10	0.20 × 0.15 × 0.10	0.60 × 0.40 × 0.20
crystal system	monoclinic	orthorhombic	triclinic	monoclinic	monoclinic
space group	*C*2/*c* (no. 15)	*Pccn* (no. 56)	$P\bar{1}$ (no. 2)	*P*2/*c* (no. 13)	$P2_1/c$ (no. 14)
a, Å	27.682(12)	14.579(5)	9.08(2)	10.962(6)	9.714(3)
b, Å	5.901(3)	25.909(6)	16.06(4)	9.650(6)	15.320(6)
c, Å	16.000(8)	11.798(3)	16.68(4)	20.748(14)	32.192(9)
α, °	90	90	114.83(7)	90	90
β, °	107.928(16)	90	96.05(11)	93.83(2)	91.107(11)
γ, °	90	90	100.27(7)	90	90
V, Å^3	2487(2)	4457(2)	2129(9)	2190(2)	4790(3)
ρ_{calcd}, g cm^{-3}	1.480	1.385	1.456	1.448	1.324
Z	4	4	2	2	4
T, K	123(2)	123(2)	123(2)	123(2)	123(2)
μ(Mo-Kα), mm^{-1}	0.928	0.893	0.577	0.567	0.519
no. of reflns	11280	25380	16009	18783	41199
no. of unique reflns	2846	5014	9022	4974	10902
variables	179	303	592	308	612
$\lambda_{Mo-K\alpha}$, Å	0.71075	0.71075	0.71075	0.71075	0.71075
R_1 ($I > 2\sigma(I)$)	0.0495	0.0507	0.1210	0.0951	0.0553
wR_2 ($I > 2\sigma(I)$)	0.0896	0.1108	0.2802	0.2002	0.1370
GOF	1.064	1.026	0.981	1.035	0.984
$\Delta\rho_{max}$, $\Delta\rho_{min}$ (e Å^{-3})	0.581, –0.517	0.415, –0.644	0.988, –1.088	1.055, –0.956	0.731, –0.858

	**2f**
formula	$C_{58}H_{58}N_4O_{16}Cu\cdot CH_2Cl_2\cdot 2CH_4O$
fw	1279.63
crystal size, mm	0.40 × 0.20 × 0.10
crystal system	monoclinic
space group	$P2_1/c$ (no. 14)
a, Å	11.915(4)
b, Å	21.393(5)
c, Å	12.778(3)
α, °	90
β, °	108.825(11)
γ, °	90
V, Å^3	3082.9(14)
ρ_{calcd}, g cm^{-3}	1.378
Z	2
T, K	123(2)
μ(Mo-Kα), mm^{-1}	0.515
no. of reflns	23931
no. of unique reflns	7069
variables	384
$\lambda_{Mo-K\alpha}$, Å	0.71075
R_1 ($I > 2\sigma(I)$)	0.0446
wR_2 ($I > 2\sigma(I)$)	0.1079
GOF	1.055
$\Delta\rho_{max}$, $\Delta\rho_{min}$ (e Å^{-3})	0.332, –0.505

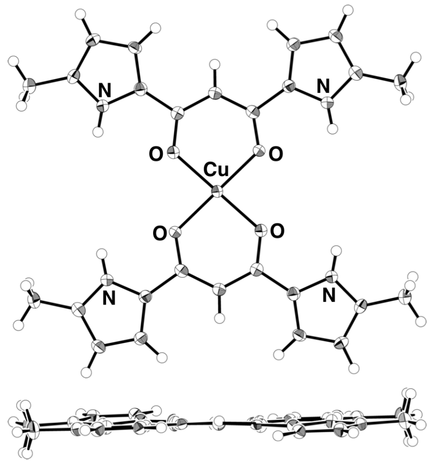

Figure S7 Ortep drawing of molecular structure (top and side views) of **1a** revealed by single-crystal X-ray analysis. Anisotropic displacement ellipsoids are scaled to the 50% probability level. Solvent molecules are omitted for clarity.

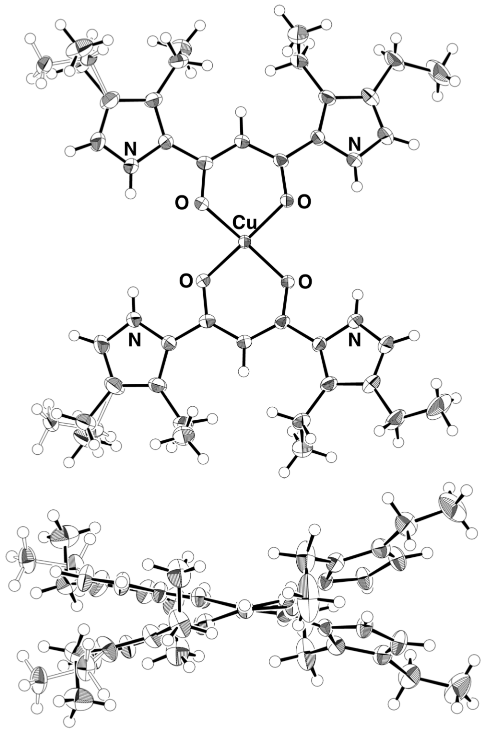

Figure S8 Ortep drawing of molecular structure (top and side views) of **1b** revealed by single-crystal X-ray analysis. Anisotropic displacement ellipsoids are scaled to the 50% probability level. Solvent molecules are omitted for clarity. One of the ethyl units has a disordered structure in the ratio of 72 (black bond) : 28 (white bond).

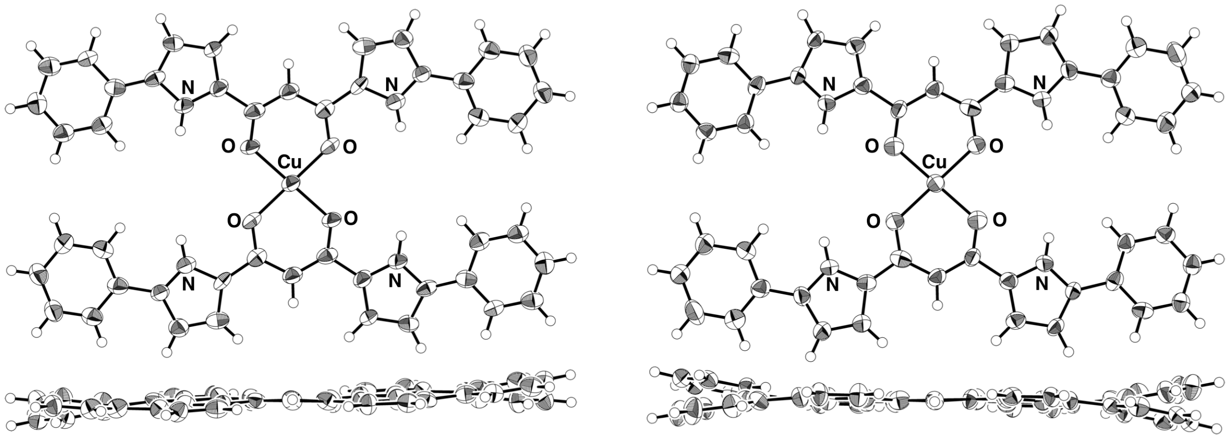

Figure S9 Ortep drawing of molecular structure (top and side views) of **2a** as two independent structures revealed by single-crystal X-ray analysis. Anisotropic displacement ellipsoids are scaled to the 50% probability level. Solvent molecules are omitted for clarity.

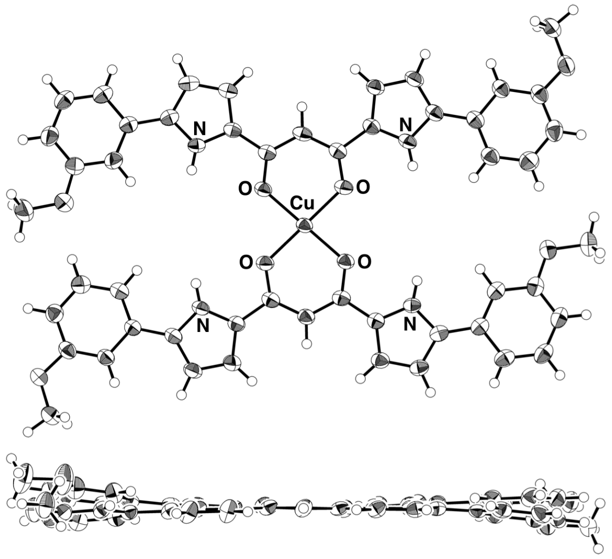

Figure S10 Ortep drawing of molecular structure (top and side views) of **2b** revealed by single-crystal X-ray analysis. Anisotropic displacement ellipsoids are scaled to the 50% probability level. Solvent molecules are omitted for clarity.

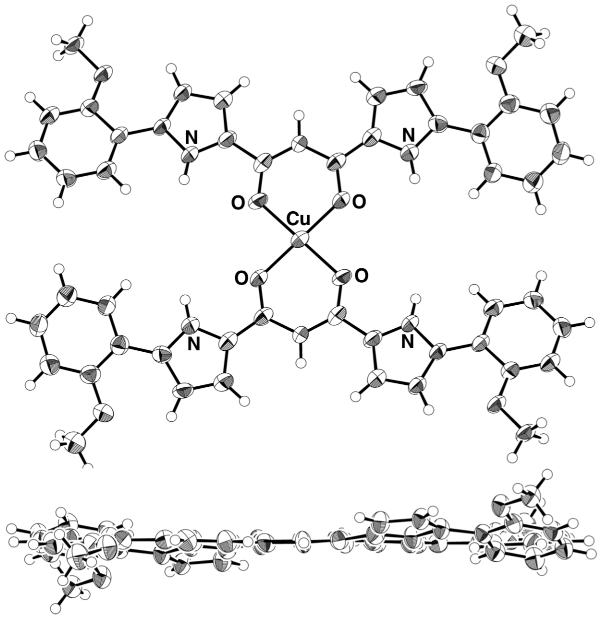

Figure S11 Ortep drawing of molecular structure (top and side views) of **2c** revealed by single-crystal X-ray analysis. Anisotropic displacement ellipsoids are scaled to the 50% probability level. Solvent molecules are omitted for clarity.

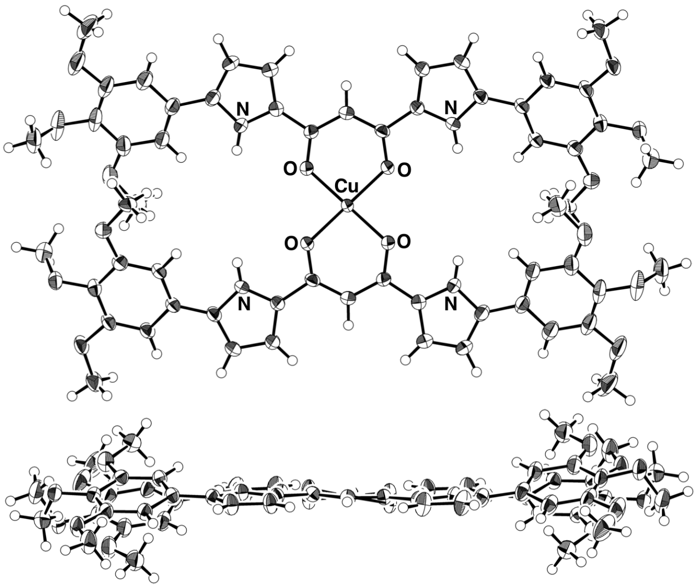

Figure S12 Ortep drawing of molecular structure (top and side views) of **2f** revealed by single-crystal X-ray analysis. Anisotropic displacement ellipsoids are scaled to the 50% probability level. Solvent molecules are omitted for clarity.

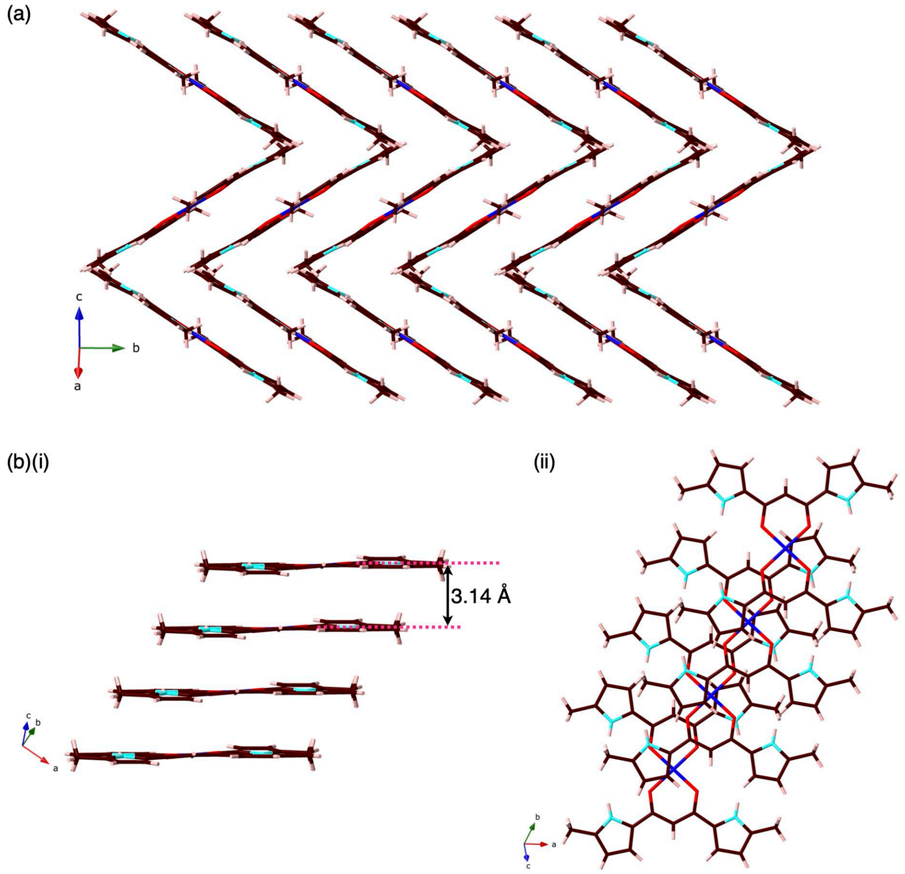

Figure S13 Packing diagrams of **1a** as (a) columnar stacking structures and (b)(i) side and (ii) top views of columnar structure based on stacking of **1a** with a stacking distance of 3.14 Å between dipyrrolyldiketone–Cu^{II} complex unit (31 atoms). **1a** showed a planar geometry with a mean-plane deviation of 0.065(2) Å (31 atoms). Atom color code: brown, pink, cyan, red, and blue refer to carbon, hydrogen, nitrogen, oxygen, and copper, respectively. Solvent molecules are omitted for clarity.

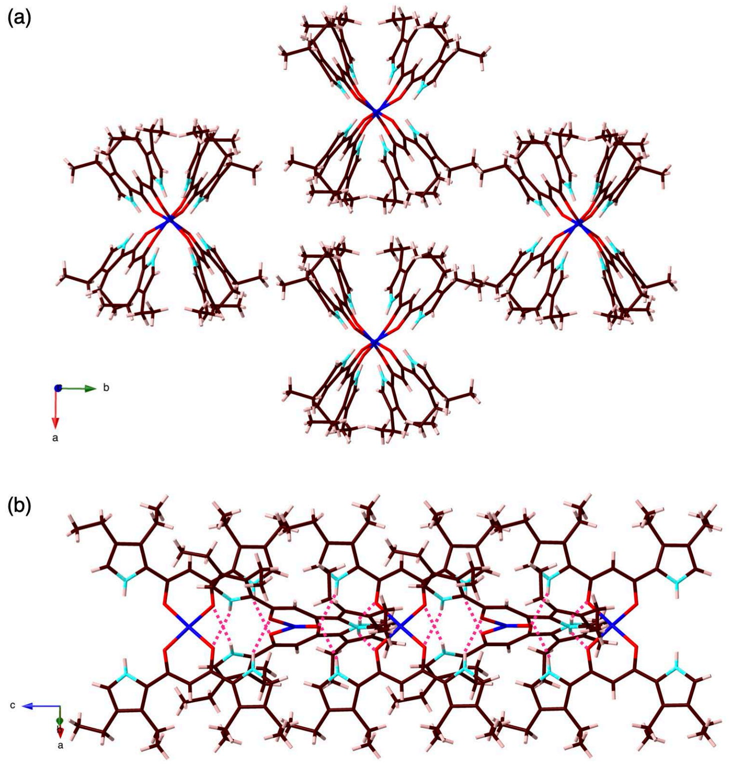

Figure S14 Packing diagrams of **1b** as (a) view from *a*-axis and (b) side view of the hydrogen-bonding 1D-arranged structure extracted from (a). Atom color code: brown, pink, cyan, red, and blue refer to carbon, hydrogen, nitrogen, oxygen, and copper, respectively. Solvent molecules are omitted for clarity.

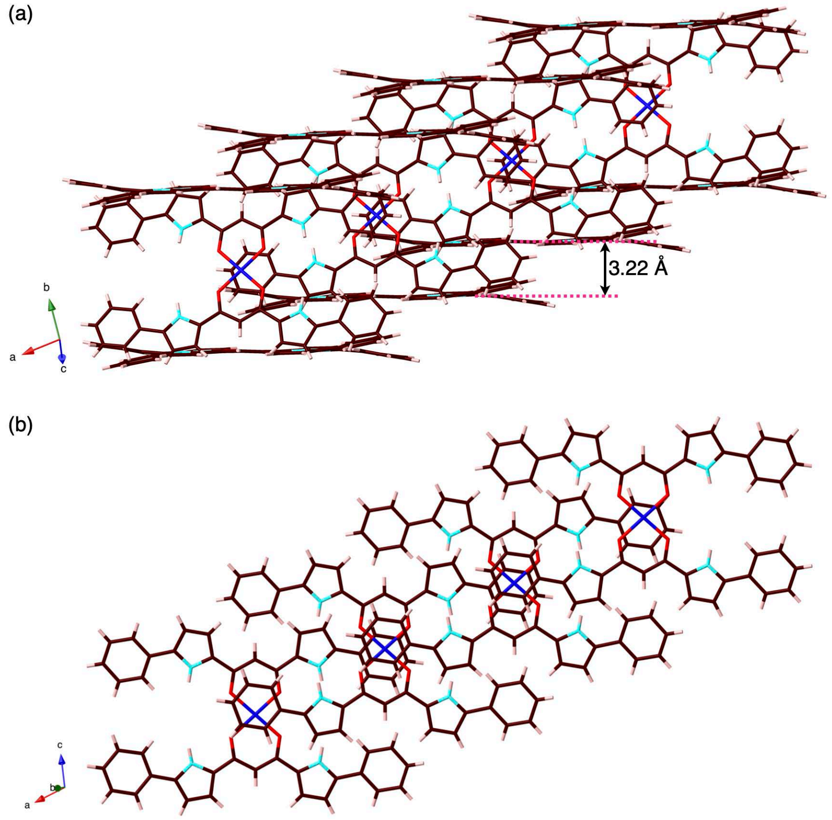

Figure S15 Packing diagrams of **2a** as (a) columnar stacking structures and (b) top view of columnar structure based on stacking of **2a** with a stacking distance of 3.22 Å between aryl-substituted dipyrrolyldiketone–Cu^{II} complex unit (55 atoms). **2a** showed a planar geometry with a mean-plane deviation of 0.041(4)/0.056(7) Å (55 atoms). Atom color code: brown, pink, cyan, red, and blue refer to carbon, hydrogen, nitrogen, oxygen, and copper, respectively. Solvent molecules are omitted for clarity.

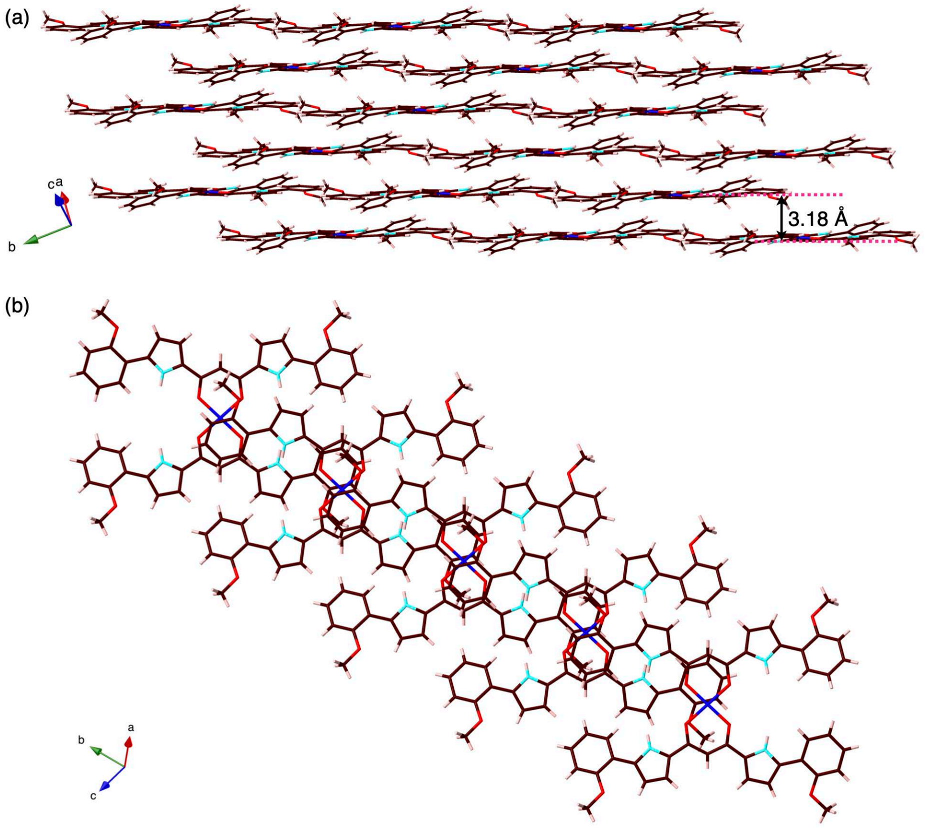

Figure S16 Packing diagrams of **2b** as (a) columnar stacking structures and (b) top view of columnar structure based on stacking of **2b** with a stacking distance of 3.18 Å between aryl-substituted dipyrrolyldiketone–Cu^{II} complex unit (55 atoms). **2b** showed a planar geometry with a mean-plane deviation of 0.127(4) Å (55 atoms). Atom color code: brown, pink, cyan, red, and blue refer to carbon, hydrogen, nitrogen, oxygen, and copper, respectively. Solvent molecules are omitted for clarity.

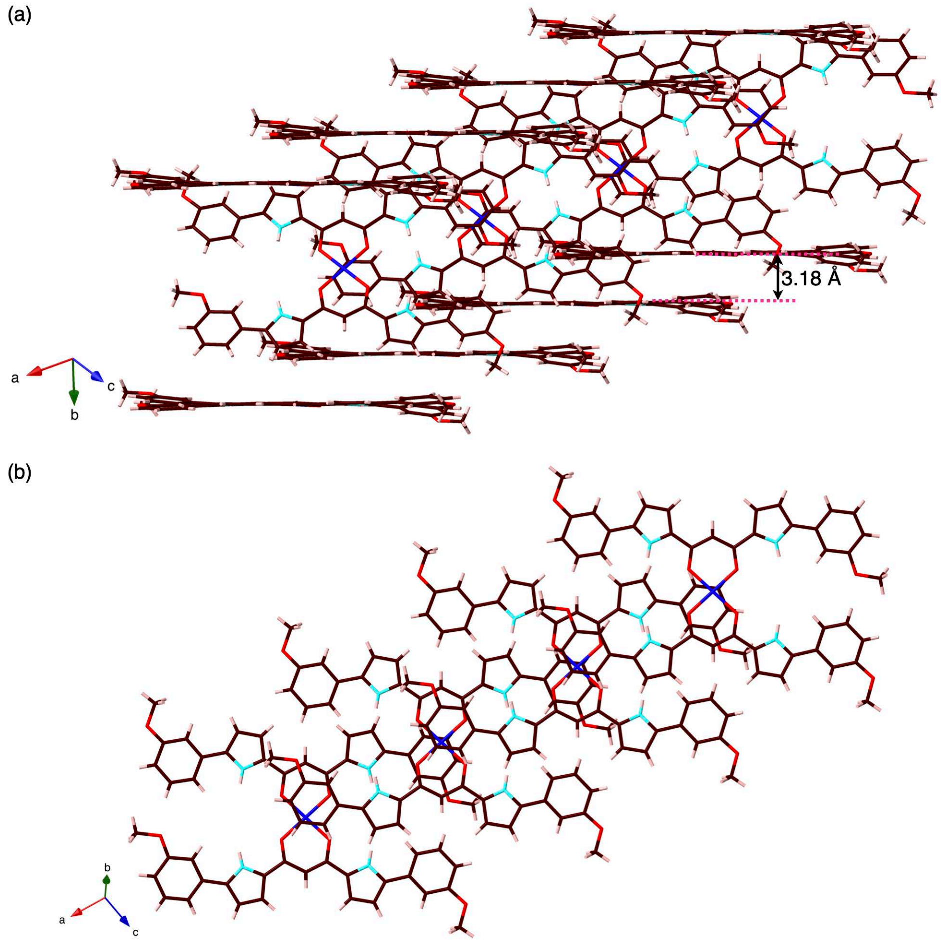

Figure S17 Packing diagrams of **2c** as (a) columnar stacking structures and (b) top view of columnar structure based on stacking of **2c** with a stacking distance of 3.18 Å between aryl-substituted dipyrrolyldiketone–Cu^{II} complex unit (55 atoms). **2c** showed a planar geometry with a mean-plane deviation of 0.029(2) Å (55 atoms). Atom color code: brown, pink, cyan, red, and blue refer to carbon, hydrogen, nitrogen, oxygen, and copper, respectively. Solvent molecules are omitted for clarity.

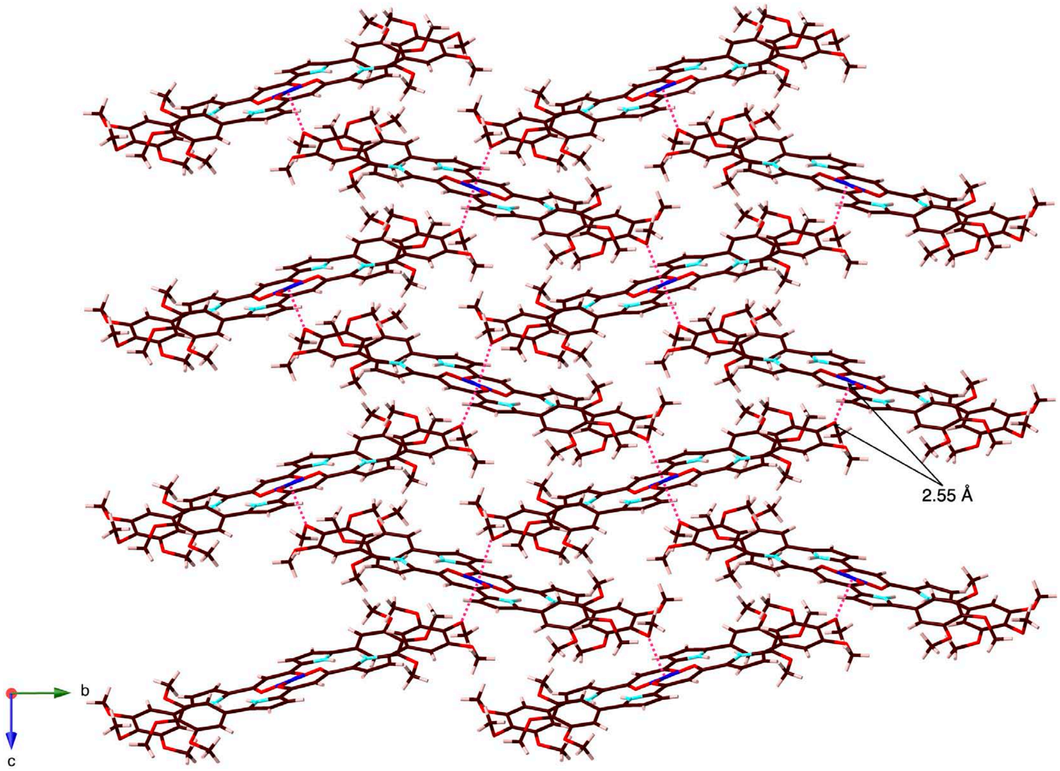

Figure S18 Packing diagram of **2f**. **2f** showed a planar geometry with a mean-plane deviation of 0.091(2) Å (55 atoms). *p*-Methoxy oxygen was coordinated to Cu^{II} of the neighboring complex with the O···Cu^{II} distance of 2.547(8) Å. Atom color code: brown, pink, cyan, red, and blue refer to carbon, hydrogen, nitrogen, oxygen, and copper, respectively. Solvent molecules are omitted for clarity.

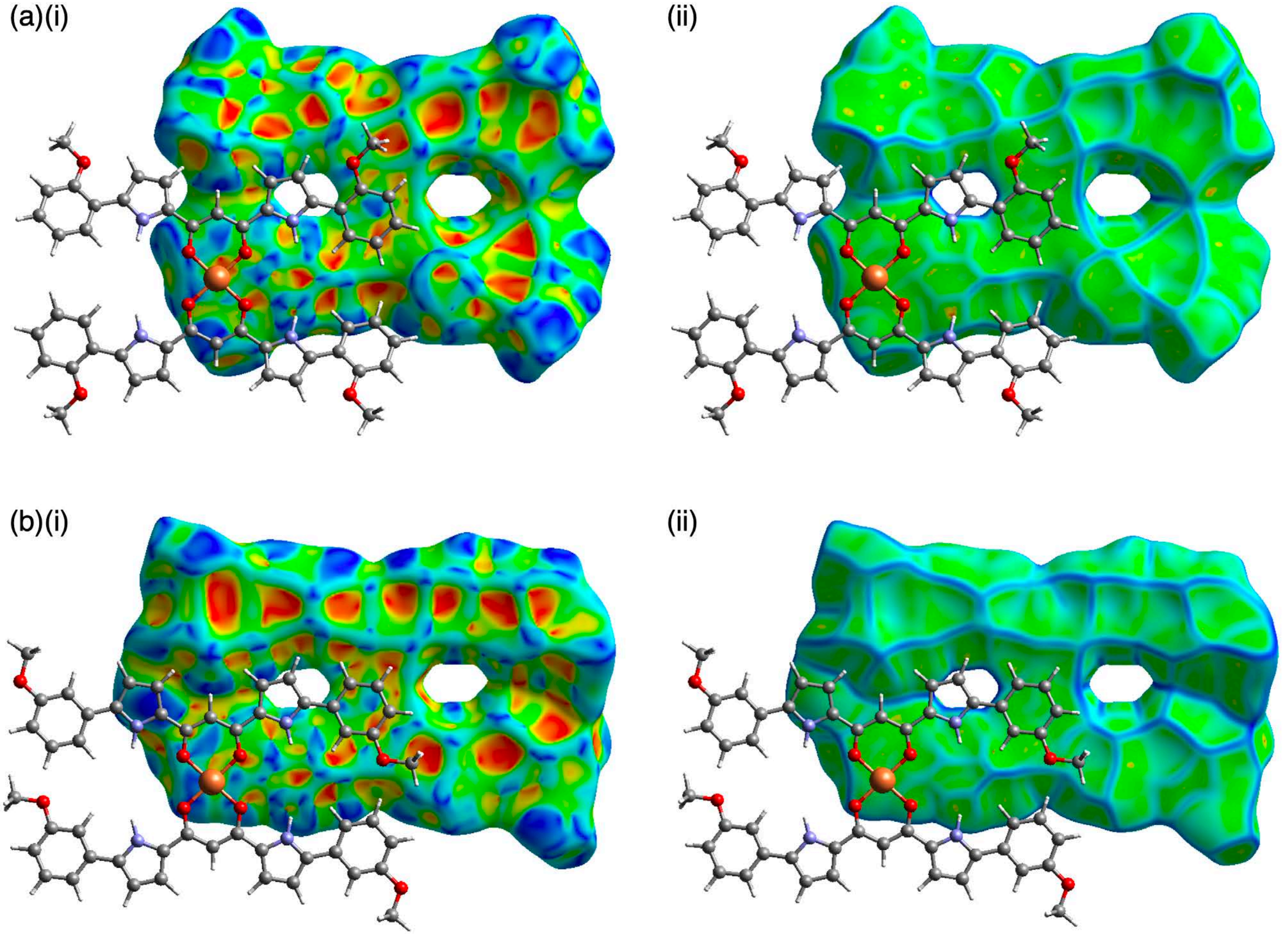

Figure S19 Hirshfeld surfaces[S1] of (a) **2b** and (b) **2c** mapped over (i) shape-index and (ii) curvedness properties. Solvent molecules are omitted for clarity. The surfaces of the aryl rings of **2b**,**c** showed the red and blue triangles arranged in bow-tie shapes on the shape-index surface and flat region on the curvedness surface, indicating the characteristic mapping pattern for the stacking of planar units including aryl rings.[S2,3] Color code: gray, white, blue, red, and orange refer to carbon, hydrogen, nitrogen, oxygen, and copper, respectively.

3. Theoretical studies

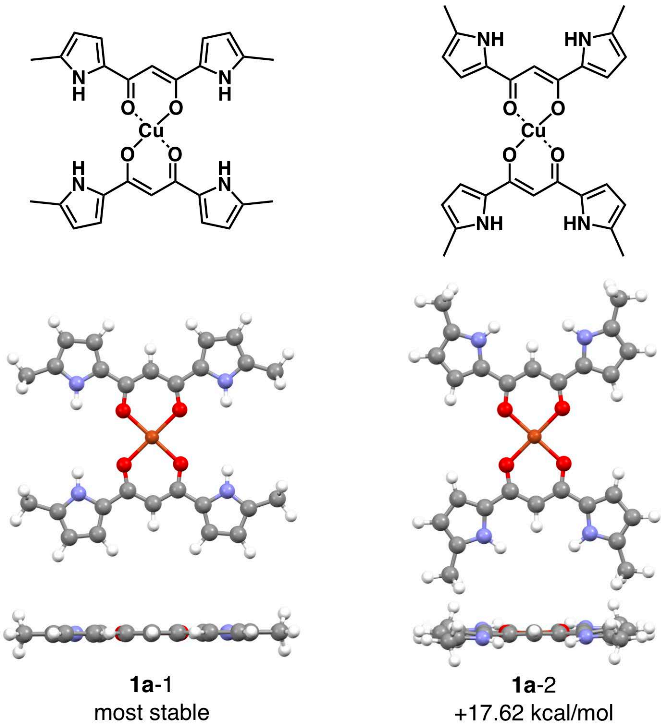

Figure S20 Optimized structures (top and side views) of **1a** at B3LYP/6-31G(d,p) for C, H, N, and O and B3LYP/LanL2DZ for Cu.

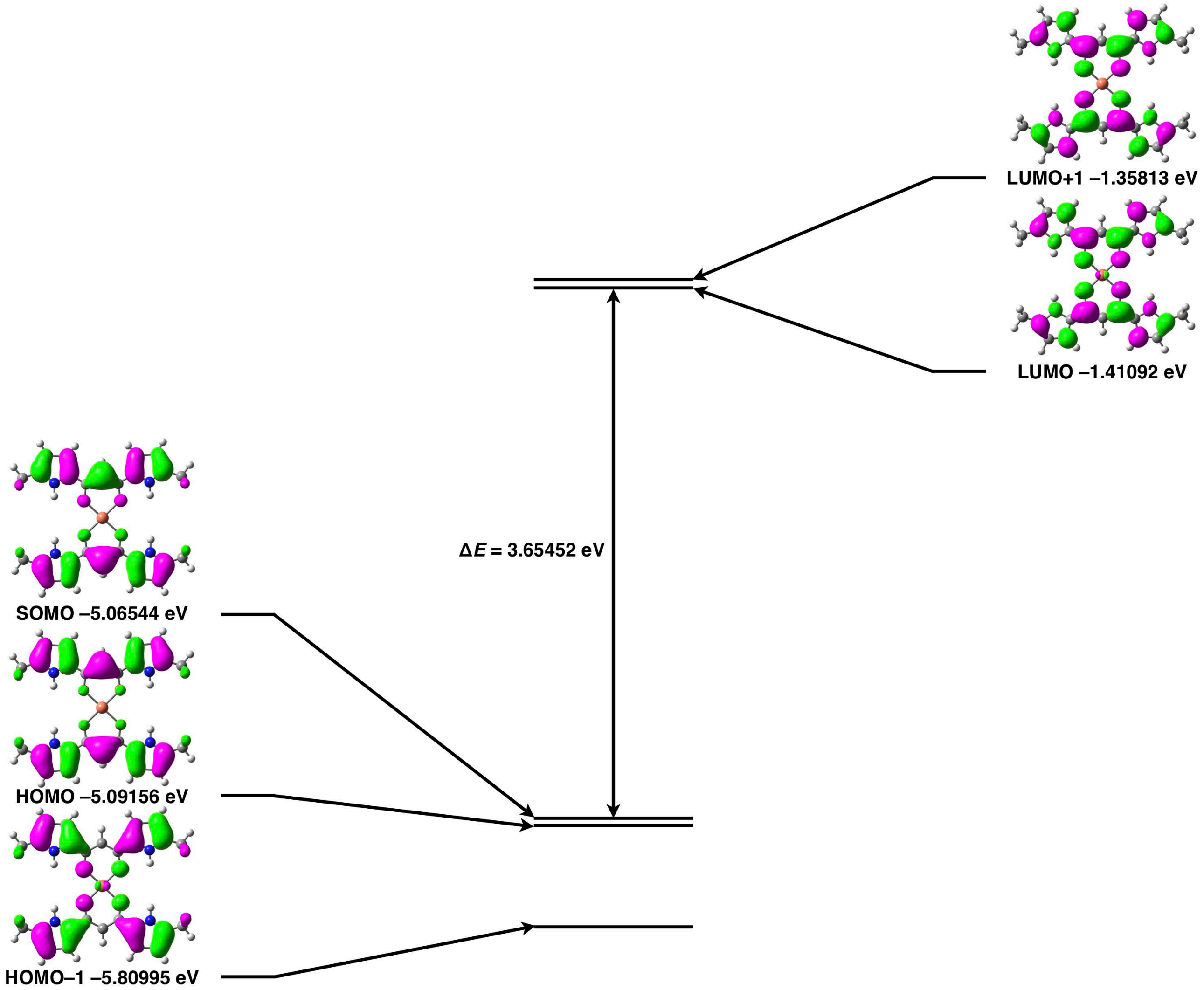

Figure S21 Molecular orbitals (HOMO/LUMO) of **1a** estimated at B3LYP/6-31G(d,p) for C, H, N, and O and B3LYP/LanL2DZ for Cu. The details of the SOMO localized at the dipyrrolyldiketone unit will be reported elsewhere.

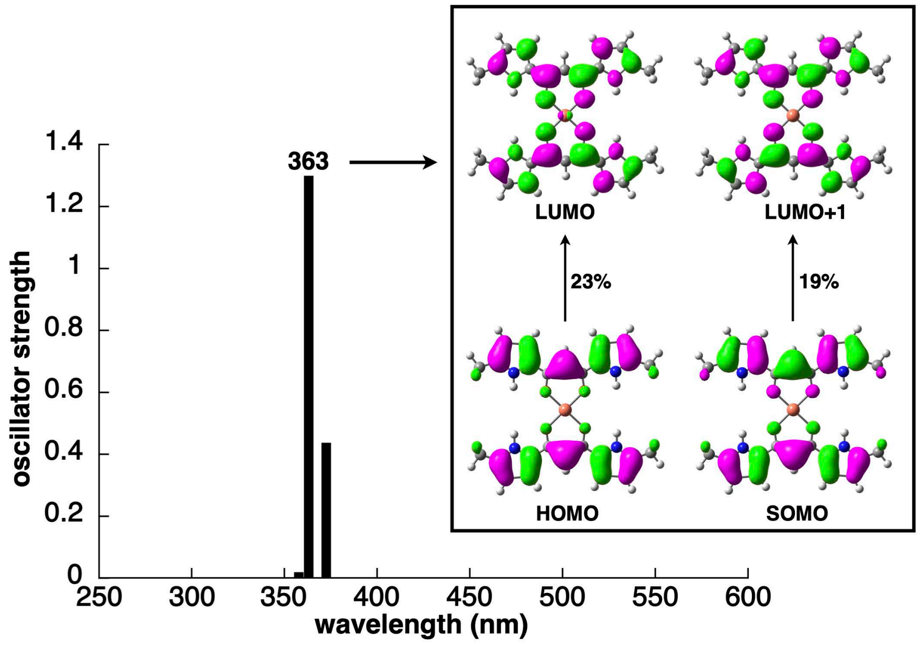

Figure S22 TD-DFT-based UV/vis absorption stick spectra of **1a** with the transitions correlated with MOs estimated at B3LYP/6-31G(d,p) for C, H, N, and O and B3LYP/LanL2DZ for Cu.

Cartesian Coordination of 1a-1

-1722.5982721 hartree

C,-4.7248401108,-3.5963543123,-0.058493131
C,4.724840115,3.5964202678,0.0552028112
C,-4.1817218052,-4.877588316,-0.0789921039
C,4.1817226346,4.8776449514,0.076295644
C,-2.7741641685,-4.7544221177,-0.0763955729
C,2.7741649172,4.7544605324,0.0748820941
C,-2.4745775869,-3.3945302776,-0.0544138536
C,2.4745776438,3.3945661004,0.0530685311
C,-1.2366317267,-2.6240824716,-0.0417228536
C,1.2366315053,2.6241039312,0.0413138147
C,-0.0001122914,-3.2850081222,-0.0518995338
C,0.0001123062,3.2850167247,0.0523190166
C,1.2364530021,-2.624179273,-0.0409400693
C,-1.2364531945,2.6241757867,0.0421380385
C,2.4743458198,-3.3947257469,-0.0527151636
C,-2.4743457822,3.3947073619,0.0548751698
C,2.7738381283,-4.7546413363,-0.0745560315
C,-2.7738371778,4.7546200029,0.0769142248
C,4.181386972,-4.8779257151,-0.0758686407
C,-4.1813860489,4.8778878046,0.079309225
C,4.7245941097,-3.5967354562,-0.0549873811
C,-4.724594004,3.5966882065,0.0590319666
Cu,-0.0000004,0.000004675,0.0001827344
H,-4.7529299962,-5.7957719658,-0.0940649189
H,4.7529318196,5.7958349049,0.0909432341
H,-2.0570057796,-5.5626912551,-0.089126626
H,2.0570070103,5.5627193173,0.0882791431
H,-0.0001495684,-4.3667844041,-0.069080382
H,0.0001498792,4.3667923545,0.0695387933
H,2.0566225493,-5.5628485553,-0.0879411484
H,-2.0566210696,5.5628372134,0.0896502127
H,4.7525335255,-5.7961559861,-0.0904353464
H,-4.7525323445,5.7961124466,0.094238375
H,-3.7092465023,-1.7113700427,-0.0278690808
H,3.7092453344,1.7114244139,0.0253284537
H,3.7091330128,-1.711669595,-0.0250519325
H,-3.7091339247,1.7116324804,0.028430037
N,-3.6778797036,-2.7202308457,-0.0439535406
N,3.6778792635,2.7202827897,0.0415689717
N,3.6776946402,-2.7205262367,-0.0412618393
N,-3.6776950918,2.7204915026,0.0444892569
O,-1.401306533,-1.3411134344,-0.021249918
O,1.4013058004,1.3411351398,0.0208176355
O,1.4012136972,-1.34122184,-0.0204829872
O,-1.4012142835,1.341219007,0.0216400096
C,6.1496471792,3.1440920707,0.046931989
H,6.3885661461,2.5245844605,0.9204371941
H,6.3884235272,2.5562399351,-0.8482336397
H,6.8161717996,4.0093225829,0.0623547772
C,-6.1494326232,3.1444374561,0.0521150677
H,-6.3890962972,2.5566130358,-0.8428319051
H,-6.3875505479,2.5249285423,0.9258377742
H,-6.8158958942,4.0097035624,0.0681852057
C,-6.1496476119,-3.1440064589,-0.0514644455
H,-6.3893485862,-2.5566035263,0.8437511071
H,-6.3876443372,-2.5240527974,-0.9249026923
H,-6.8161701268,-4.0092177421,-0.0680170337
C,6.1494320173,-3.1445043048,-0.0467655344
H,6.3882543849,-2.5246800103,-0.9200710643
H,6.3883877593,-2.5570086502,0.8485876054
H,6.8158980134,-4.0097723051,-0.0626187342

Cartesian Coordination of 1a-2

-1722.570196 hartree

C,-3.8083896784,5.068131325,-0.3328953434
C,3.9015346397,-5.2929528252,0.2012731639
C,-4.5883522917,3.944339084,-0.0976686218
C,4.681365235,-4.1690837862,-0.0340263664
C,-3.7257651083,2.8280706328,-0.0049603671
C,3.8187153407,-3.0528058403,-0.1260045755
C,-2.4245697246,3.2812129361,-0.1758912185
C,2.517605422,-3.5060072222,0.0454430966
C,-1.1820938058,2.5109057359,-0.1492747918
C,1.2751174241,-2.7356897053,0.0196608268
C,0.0643218479,3.1682423548,-0.1177611364
C,0.0286611849,-3.3929864623,-0.0112777168
C,1.3041551421,2.4978420071,-0.1232914024
C,-1.2111489073,-2.7225600804,-0.00481378
C,2.5549776415,3.2549789632,-0.1236601016
C,-2.461983162,-3.4796797582,-0.003597942
C,3.847537291,2.7879000671,0.0733639838
C,-3.7546332422,-3.0126323519,-0.2000867361
C,4.7236360873,3.8949940507,-0.0006152786
C,-4.6307150703,-4.1196925625,-0.1253721763
C,3.9606066424,5.0272079979,-0.2505679506
C,-3.8675816476,-5.2518469584,0.1245231748
Cu,0.0465169723,-0.1123856642,-0.0643400787
H,-5.6656266912,3.9446140296,-0.0029099707
H,5.7585925324,-4.1693124774,-0.1293154641
H,-3.9896771524,1.7976448428,0.1802569567
H,4.0825286903,-2.0223275969,-0.3110679892
H,0.0688351492,4.2471450481,-0.0107093529
H,0.0240448271,-4.4718728463,-0.118473715
H,4.0966617432,1.7545730854,0.2628474103
H,-4.0038110642,-1.9793555392,-0.3897728703
H,5.7986953055,3.8837361608,0.1160867712
H,-5.7058311542,-4.1084586192,-0.2415530671
H,-1.7222354726,5.2444806921,-0.604573632
H,1.8155250022,-5.469414821,0.4739287342
H,1.8824001192,5.2260421434,-0.5642830026
H,-1.7892370347,-5.4506135413,0.4373354316
N,-2.5017169951,4.6538029545,-0.3648133695
N,2.594879219,-4.878642605,0.2339924587
N,2.6505567716,4.6268053946,-0.3096868581
N,-2.5574870428,-4.8514544198,0.1828343804
O,-1.3405803223,1.2367185269,-0.1529858568
O,1.4336448199,-1.4615037416,0.0235041059
O,1.4492511435,1.2220517747,-0.1239934805
O,-1.3562255101,-1.4467663818,-0.0038724199
C,4.3641701474,6.4538065727,-0.4431529446
H,4.0865621276,6.830826004,-1.4359325761
H,3.9043569377,7.1164920977,0.300877299
H,5.4479301533,6.5494022449,-0.3462900694
C,-4.192756563,6.4987146892,-0.534953271
H,-3.7409803738,7.1573441725,0.2175516702
H,-3.8912107283,6.8715917565,-1.5223023995
H,-5.2771670332,6.6059534742,-0.4600438149
C,-4.2710776798,-6.6783686975,0.3178173202

H,-3.8112387512,-7.3413958289,-0.4258899804
H,-3.9934491146,-7.0548888482,1.3107829621
H,-5.3548341711,-6.7740663857,0.2210100898
C,4.286008603,-6.7235988954,0.4026884691
H,3.9847389298,-7.0968530363,1.3899782729
H,3.8340522423,-7.3819631843,-0.3499416964
H,5.3704022174,-6.830774136,0.3274462467

5. Assembled behaviors

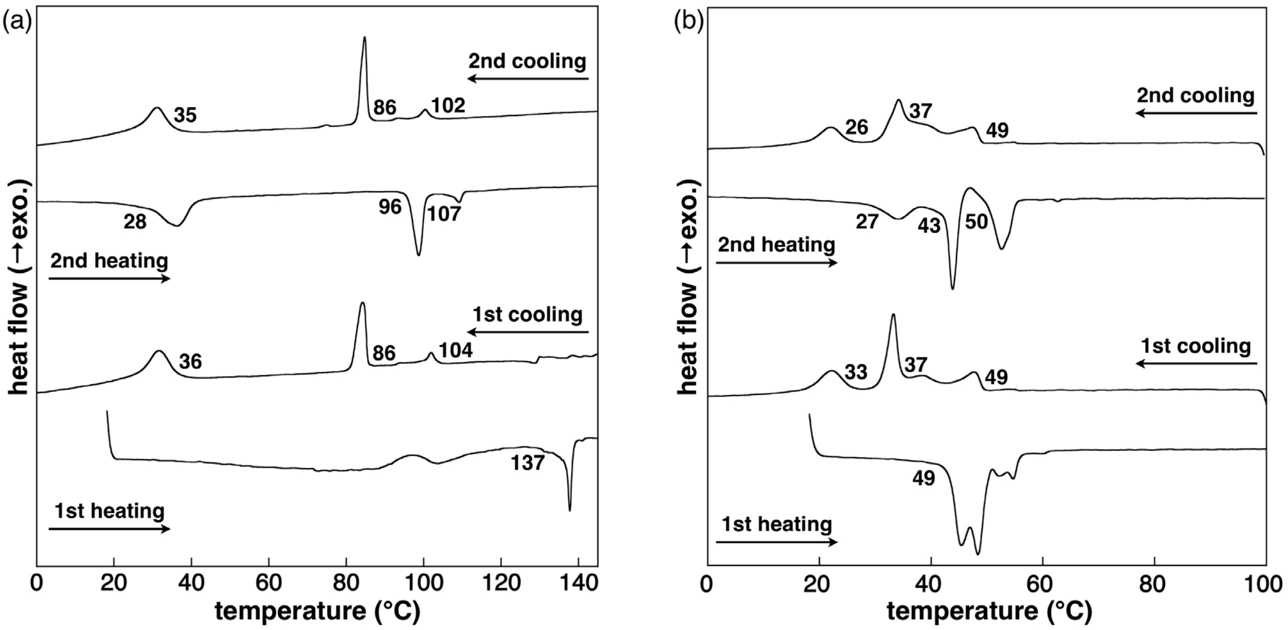

Figure S23 DSC thermograms of (a) **3a** and (b) **3b**. Onset temperatures (°C) of phase transitions are labeled although some peaks are weak.

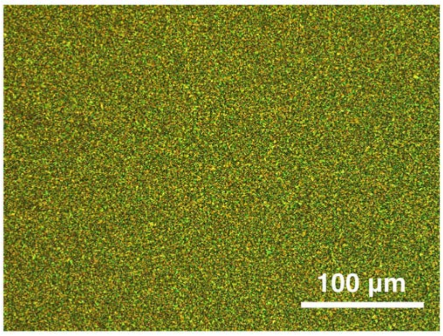

Figure S24 POM texture of **3a** at 90 °C upon cooling. POM texture of **3b** was not clearly observed in the mesophase.

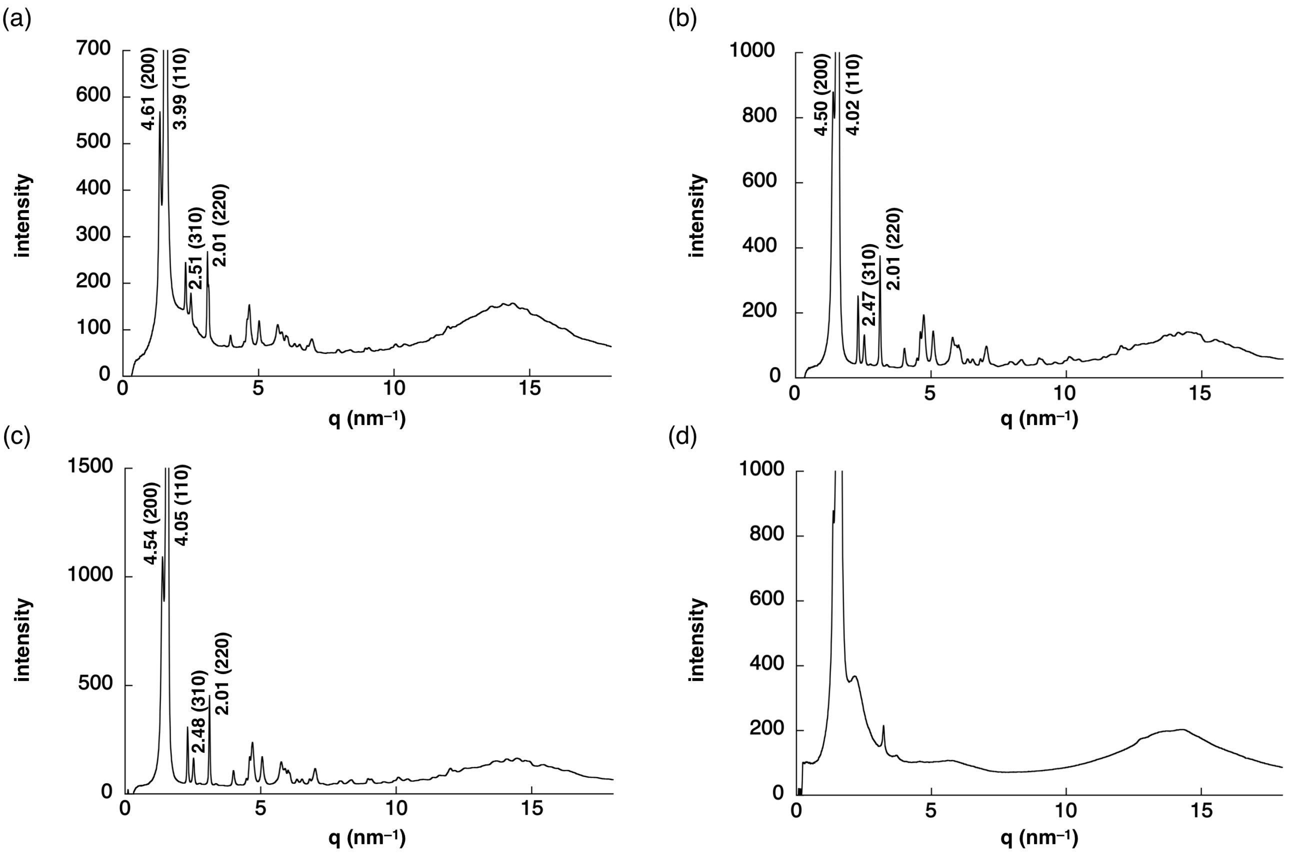

Figure S25 XRD patterns of **3a** at (a) 90 °C, (b) 70 °C, (c) 80 °C, and (d) 105 °C upon (a,b) 1st cooling and (c,d) 2nd heating. Complicated peaks were observed in (a–c), suggesting the crystalline state.

Table S2 Summary of XRD data of **3a**. The peaks which can be indexed are represented.

	q (nm^{-1})	*d*-spacing (nm)	ratio	ratio (calc.)	*hkl*
(a) 90 °C (1st cooling)	1.36	4.61	1.00	1.000	100
Col_r (*P*2/*a*)	1.57	3.99	0.86	0.866	110
$a = 9.22$ nm, $b = 4.43$ nm, $c = 0.39$ nm	2.51	2.51	0.54	0.548	310
$M = 2693.8$, $Z = 4$ ($\rho = 1.11$)	3.12	2.01	0.44	0.433	220
	16.3	0.39	–	–	001
(b) 70 °C (1st cooling)	1.40	4.50	1.00	1.000	100
Col_r (*P*2/*a*)	1.56	4.02	0.89	0.893	110
$a = 9.00$ nm, $b = 4.49$ nm, $c = 0.41$ nm	2.54	2.47	0.55	0.554	310
$M = 2693.8$, $Z = 4$ ($\rho = 1.08$)	3.11	2.01	0.45	0.447	220
	15.5	0.41	–	–	001
(c) 80 °C (2nd heating)	1.38	4.54	1.00	1.000	100
Col_r (*P*2/*a*)	1.55	1.44	0.89	0.893	110
$a = 9.07$ nm, $b = 4.53$ nm, $c = 0.41$ nm	2.53	2.53	0.55	0.554	310
$M = 2693.8$, $Z = 4$ ($\rho = 1.05$)	3.11	2.01	0.44	0.446	220
	15.4	0.41	–	–	001

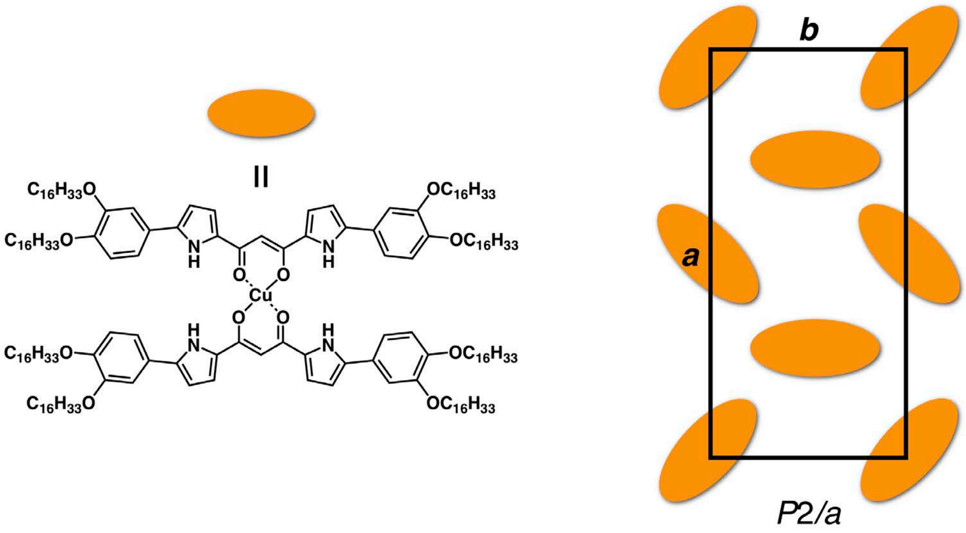

Figure S26 Possible packing model of **3a** as a Col_r structure.

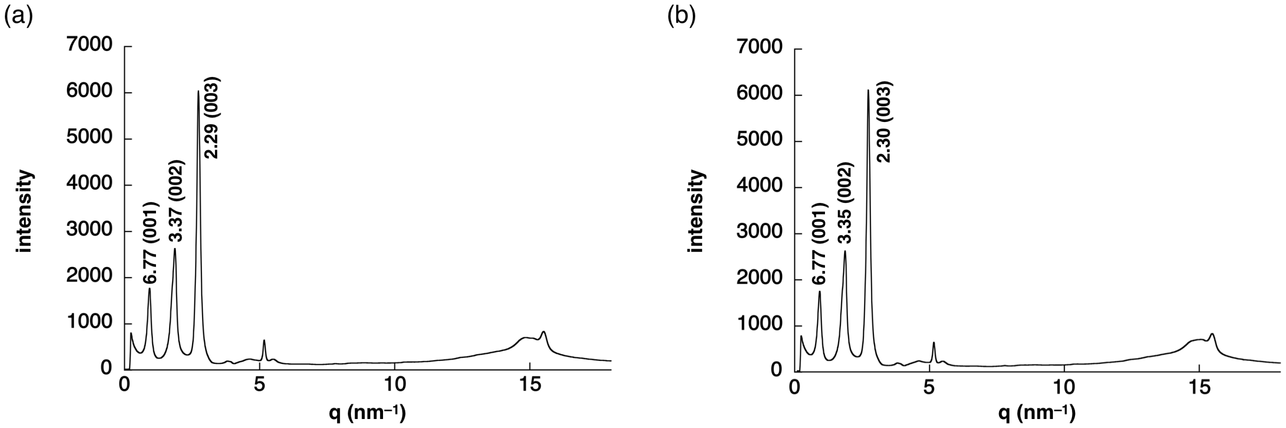

Figure S27 XRD patterns of **3b** at (a) 35 °C and (b) 38 °C upon (a) 1st cooling and (b) 2nd heating.

Table S3 Summary of XRD data of **3b**. The peaks which can be indexed are represented.

	q (nm^{-1})	*d*-spacing (nm)	ratio	ratio (calc.)	*hkl*
(a) 35 °C (1st cooling) lamellar	0.93	6.77	1.00	1.000	001
	1.87	3.37	0.50	0.500	002
	2.73	2.29	0.34	0.333	003
(b) 38 °C (2nd heating) lamellar	0.93	6.77	1.00	1.000	001
	1.86	3.35	0.50	0.500	002
	2.74	2.30	0.34	0.333	003

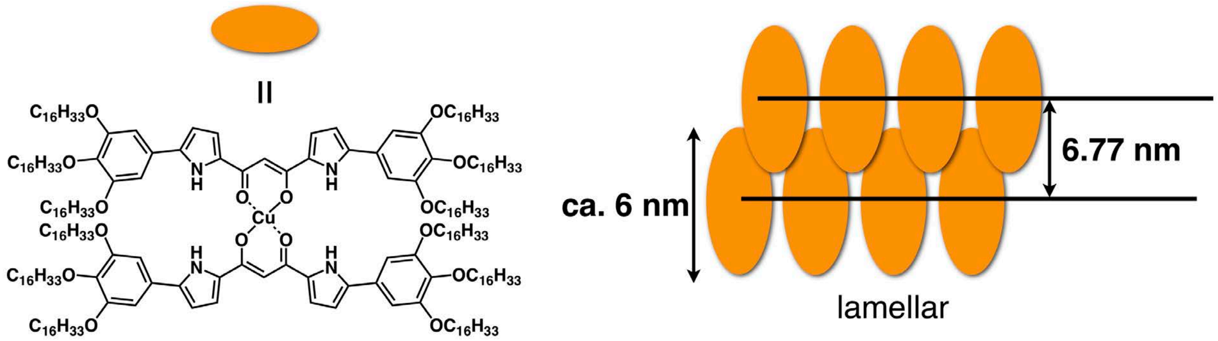

Figure S28 Possible packing model of **3b** as a lamellar structure.

References

[S1] Turner, M. J.; McKinnon, J. J.; Wolff, S. K.; Grimwood, D. J.; Spackman, P. R.; Jayatilaka, D.; Spackman, M. A. *CrystalExplorer17*, University of Western Australia, 2017.

[S2] McKinnon, J. J.; Spackman, M. A.; Mitchell, A. S. Novel tools for visualizing and exploring intermolecular interactions in molecular crystals. *Acta Crystallogr. Sect. B* **2004**, *60*, 627–668.

[S3] Tan, S. L.; Jotani, M. M.; Tiekink, E. R. T. Utilizing Hirshfeld surface calculations, non-covalent interaction (NCI) plots and the calculation of interaction energies in the analysis of molecular packing. *Acta Crystallogr. Sect. E* **2019**, *75*, 308–318.
